# Position Statement of the Brazilian Cardiology Society and the Brazilian Society of Hemodynamics and Interventional Cardiology on Training Centers and Professional Certification in Hemodynamics and Interventional Cardiology - 2020

**DOI:** 10.36660/abc.20190841

**Published:** 2020-01

**Authors:** José Airton de Arruda, Viviana de Mello Guzzo Lemke, José Mariani Júnior, Adriano Henrique Pereira Barbosa, Alexandre Schaan de Quadros, Carlos Augusto Cardoso Pedra, Cristiano de Oliveira Cardoso, Ênio Eduardo Guérios, Henrique Barbosa Ribeiro, Luiz Antonio Gubolino, Maurício Cavalieri Machado, Mauricio Jaramillo Hincapie, Nelson Antonio Moura de Araujo, Raul Ivo Rossi Filho, Ricardo Alves da Costa, Silvio Gioppato

**Affiliations:** 1 Grupo Meridional de Saúde, Vitória, ES - Brazil; 2 Cardiocare Clínica Cardiológica, Curitiba, PR - Brazil; 3 Hospital Israelita Albert Einstein, São Paulo, SP - Brazil; 4 Santa Casa de São Paulo, São Paulo, SP - Brazil; 5 Instituto do Coração (InCor) do Hospital das Clínicas da Faculdade de Medicina da Universidade de São Paulo (HCFMUSP), São Paulo, SP - Brazil; 6 Escola Paulista de Medicina (EPM) da Universidade Federal de São Paulo (UNIFESP), São Paulo, SP - Brazil; 7 Sociedade Brasileira de Hemodinâmica e Cardiologia Intervencionista (SBHCI), São Paulo, SP - Brazil; 8 Instituto Dante Pazzanese de Cardiologia, São Paulo, SP - Brazil; 9 Hospital do Coração (HCor), São Paulo, SP - Brazil; 10 Instituto de Cardiologia do Rio Grande do Sul, Porto Alegre, RS - Brazil; 11 CINECORS Cardiologia LTDA, Porto Alegre, RS - Brazil; 12 Hospital de Clínicas da Universidade Federal do Paraná (UFPR), Curitiba, PR - Brazil; 13 Hospital Pilar, Curitiba, PR - Brazil; 14 Instituto do Coração de Piracicaba (INCORPI), Piracicaba, SP - Brazil; 15 Hospital dos Fornecedores de Cana de Piracicaba, Piracicaba, SP - Brazil; 16 Instituto do Coração de Rio Preto, São José do Rio Preto, SP - Brazil; 17 Hospital Beneficência Portuguesa de São José do Rio Preto, São José do Rio Preto, SP - Brazil; 18 Santa Casa de Limeira, Limeira, SP - Brazil; 19 Hospital Unimed Americana, Americana, SP - Brazil; 20 AUSTA hospital, São José do Rio Preto, SP - Brazil; 21 Hospital Governador Israel Pinheiro, Belo Horizonte, MG - Brazil; 22 Hospital do Coração do Brasil, Brasília, DF - Brazil; 23 Hospital Brasília, Brasília, DF - Brazil; 24 Pronto-Socorro Cardiológico Universitário de Pernambuco (PROCAPE) da Universidade de Pernambuco (UPE), Recife, PE - Brazil; 25 Hospital das Clínicas da Universidade Federal de Pernambuco, Recife, PE - Brazil; 26 Hospital Unimed Recife III, Recife, PE - Brazil; 27 Universidade Estadual de Campinas (UNICAMP), Campinas, SP - Brazil; 28 Hospital Vera Cruz, Campinas, SP - Brazil

**Table:** 

Declaration of potential conflict of interests of authors/collaborators of the Position Statement of the Brazilian Cardiology Society and the Brazilian Society of Hemodynamics and Interventional Cardiology on Training Centers and Professional Certification in Hemodynamics and Interventional Cardiology - 2020 If, within the last 3 years, the author/collaborator of the guideline:							
Names of guideline collaborators	Participated in clinical and/or experimental studies sponsored by pharmaceutical or equipment companies related to this guideline	Spoke at events or activities sponsored by industry related to this guideline	Was (is) a member of a board of advisors or a board of directors of a pharmaceutical or equipment industry	Participated in normative committees of scientific research sponsored by industry	Received personal or institutional funding from industry	Wrote scientific papers in journals sponsored by industry	Owns stocks in industry
Adriano Henrique Pereira Barbosa	No	No	No	No	No	No	No
Alexandre Schaan de Quadros	BMS	No	No	No	Terumo, Asahi, Biotronik, Medtronic, Boston, Abbott	No	No
Carlos Augusto Cardoso Pedra	Venus Medtech	Medtronic, Abbott	No	No	No	No	No
Cristiano de Oliveira Cardoso	No	Cannon Medical, Systems do Brasil	No	No	No	No	SafetyGRAY
Ênio Eduardo Guérios	Abbott, Lifetech	No	No	No	No	No	No
Henrique Barbosa Ribeiro	No	No	"Medtronic, ScitechMedical, Meril Lifesciences"	No	Medtronic, ScitechMedical, Meril Lifesciences, Edwards Lifesciences, Boston Scientific	Abbott, Edwards Lifesciences	No
José Airton de Arruda	No	No	ScitechMedical	No	ScitechMedical, ArtMédica	No	No
José Mariani Junior	No	No	No	No	No	No	No
Luiz Antonio Gubolino	No	No	No	No	No	No	No
Maurício Cavalieri Machado	No	No	No	No	No	No	No
Mauricio Jaramillo Hincapie	No	No	No	No	No	No	No
Nelson Antonio Moura de Araujo	No	No	No	No	No	No	No
Raul Ivo Rossi Filho	No	No	No	No	No	No	No
Ricardo Alves da Costa	No	No	No	No	No	No	No
Silvio Giopatto	No	No	No	No	No	No	No
Viviana de Mello Guzzo Lemke	No	No	No	No	No	No	No

## 1. Introduction

The first coronary angioplasty, performed by Gruntzig^1^ in 1977, marked the beginning of a revolution in cardiovascular disease treatment. Coronary angioplasty was initially considered an alternative to myocardial revascularization. With the passing of the years, accompanied by great technical-scientific advancement, percutaneous coronary intervention (PCI) has gone on to become the modality of choice when opting for mechanistic treatment of obstructive coronary disease. In this manner, coronary obstructions, with their diverse scenarios of complexity and forms of clinical presentation, are currently preferably treated by means of percutaneous techniques. Great advances in scientific knowledge and the development of increasingly less invasive techniques have also made it possible to overcome the limits of the territory of coronary circulation. The range of interventional treatments for cardiovascular and structural heart diseases is increasingly broad, and it represents a new branch of PCI, encompassing congenital and acquired heart diseases which were previously treated by traditional surgery or not even addressed. All of these enormous advances witnessed over the past decades have expanded not only the capacity but also the responsibility of interventional cardiologists within this new model of percutaneous treatments for cardiovascular diseases. The competences attributed to interventional cardiologists have gone on to include percutaneous treatment of structural heart diseases and extracardiac arterial and venous vascular territories, which, in addition to treatment of coronary obstructions, requires a broad and sophisticated process of training and certification.

As the realm of diseases that may be treated by means of percutaneous techniques expands, the processes for training, certifying, and keeping abilities up-to-date undergo a true metamorphosis for interventional cardiologists. It is thus necessary for the Brazilian Society of Hemodynamics and Interventional Cardiology (Sociedade Brasileira de Hemodinâmica e Cardiologia Intervencionista - SBHCI) to revise the stages adopted in this complex process, with the objective of guaranteeing civil society’s access to professionals with the abilities, competences, and responsibility to carry out percutaneous treatment of the diverse diseases included in this vast scenario in a proper and safe manner.

## 2. Objectives

This Position Paper is an update to the second chapter of the previous edition of the Guidelines on Quality and Professional Certification,^2^ and its objective is to offer a guide to orient professionals, training centers, and institutions acting in the area of cardiovascular intervention (Hemodynamics and Interventional Cardiology) in relation to PCI, congenital and structural heart diseases, and intervention in the extracardiac arterial and venous vascular bed.

The recommendations contained in this document are in following with the standards established by the Brazilian Cardiology Society (Sociedade Brasileira de Cardiologia - SBC) for the elaboration of position papers, guidelines, and normalizations and they comprise recommended actions that are, generally, individualized for each specific topic covered in this document.

## 3. Norms for Establishing Hemodynamics and Interventional Cardiology Training Centers

The SBHCI has always been vigilant regarding the formation of new professionals in the area, elaborating norms that guarantee the quality of services provided at diverse hospitals in diverse communities.

These position papers, which are the result of consensus between members of the commission who participated in their elaboration, are intended to update the criteria for capacitating training centers acting in the area of interventional cardiology.

Existing centers and centers that may be established in the future must comply with the following criteria in order to maintain or apply for accreditation as **SBHCI Hemodynamics and Interventional Cardiology Training Centers.**^3^

### 3.1. Basic Requirements

#### 3.1.1. Hospitals (Institutions)

A hospital which is a candidate to become a Training Center must apply to the SBHCI, and it must meet the following essential composition and resource requirements:

- Hemodynamics and interventional cardiology laboratory.
- Active cardiovascular surgery service.
- Intensive therapy unit or coronary unit.
- Clinical analysis laboratory.
- Hemotherapy service.
- Imaging services, comprising radiology, transthoracic and transesophageal Doppler echocardiography, preferably with 3-D reconstruction, Doppler ultrasound, computerized tomography (CT) and/or magnetic resonance (MR), digestive endoscopy.
- Hemodialysis service.
- Clinical specialties (cardiology, radiology, nephrology, neurology, gastroenterology, pneumology, hematology, and anesthesiology).
- Ventricular assist devices.

#### 3.1.2. Hemodynamics Laboratories

To the extent that the spectrum of interventional procedures expands, in conjunction with the complexity of “old” and new procedures, catheterization laboratories must have equipment and instruments that are compatible with this new working scenario, especially in training centers.

The supplies necessary for catheterization laboratories to function and the role of nurses, radiology technicians, and other members of the interventional cardiology teams are described in the latest guidelines on professional quality, and they are in consonance with current practice.^2^ The necessary requirements for structural constitution of a training center catheterization laboratories are described in Chart 1.

**Chart 1:** Necessary requirements for structural constitution of a training center catheterization laboratories

1.	A radiological device, adequately fixed to the ground or ceiling, and a motorized C-arm system
2.	Architecture that allows for axial projections with 40º angulation and oblique projections with 90º angulation, by electronic movement
3.	An examination table with capacity to support patients weighing up to 200 kg, plus 100 kg imposed during reanimation maneuvers
4.	A high-voltage X-ray generator with 80 kW minimum power, for rapid radiation emission, sufficient to obtain image contrast and sharpness
5.	An X-ray tube with minimal thermal capacity of 1,700,000 HU
6.	Pulsed fluoroscopy with rates of at least 30/15 pulses per second
7.	An image intensifier with the highest possible conversion factor or a flat-panel digital system
8.	High-quality digital imaging with a matrix of at least 512 × 512 × 8 bits at 30 frames per second
9.	Long-term (20-year) digital archiving in DICOM format
10.	Polygraph with a record of at least three electrocardiogram and two pressure channels
11.	Contrast injection pump
12.	An anticoagulation monitoring device by measurement of activated coagulation time
13.	Pulse oximetry
14.	Equipment for measurement of cardiac output by thermodilution
15.	Material for cardiorespiratory resuscitation and external pacemaker/defibrillator
16.	Intracardiac lead and temporary pacemaker generator
17.	Radiation protection equipment

For Training Centers that utilize more than one catheterization laboratories in different hospitals, it is mandatory for each unit to comply with the basic minimum requirements listed in this Position Paper.

#### 3.1.3. Medical Teams

Medical teams must be made up of preceptors and a coordinator, provided that they meet the following requirements:

- To have at least two preceptors, both titular members of the SBHCI for at least five years, with certificates in the area of hemodynamics and interventional cardiology provided by the SBHCI. Each preceptor should perform at least 75 cardiovascular therapeutic interventions annually and demonstrate maintenance of proficiency by sending a record of these interventions to the current SBHCI database.
- The program coordinator must be one of the preceptors, and he or she will responsible to the SBHCI for compliance with these recommendations.
- Perform at least 1,500 diagnostic cardiac catheterizations annually, proven by a declaration signed by the technician responsible for the service.
- Perform cardiovascular interventions, with at least 600 PCI yearly, proven by a declaration signed by the technician responsible for the service.
- Send the records of all cardiovascular interventions performed annually to the current SBHCI database.
- Follow the theoretical-practical program recommended in this Position Paper.
- The number of openings made available to candidates per team per year must comply with the following limits: for the minimum requisite of 600 annual angioplasties, up to two openings; for each increment of 200 PCI, one opening may be added. It is necessary that there be a ratio of one preceptor per trainee (Chart 2).
  **Chart 2:** Minimum number of procedures required for formation of interventional cardiologists

Procedure	Yearly minimum for a training center	Minimum per trainee in 2 years (first operator)
Diagnostic cardiac catheterization	1,500	400
Percutaneous coronary intervention	600	200

#### 3.1.4. Trainees

The prerequisites and obligations of trainees include a degree in Cardiology and full-time dedication to the training program, in addition to the following:^3^

- Be duly registered with the Regional Council of Medicine (Conselho Regional de Medicina - CRM) of the state where the training center is located.
- Have completed two years of medical residency in Cardiology at a service accredited by the National Medical Residency Commission (Comissão Nacional de Residência Médica - CNRM); have completed two years of internship in Cardiology in training centers recognized by the SBC or hold the title of specialist in Cardiology issued by the Brazilian Medical Association (Associação Médica Brasileira - AMB)/SBC.
- For the interventional cardiology for congenital heart diseases program, in place of the previous items, the candidate must have completed two years of medical residency in Pediatric Cardiology at a service accredited by the CNRM and have completed two years of internship in Pediatric Cardiology in training centers recognized by the SBC or hold the title of specialist in Pediatrics acting in the area of Pediatric Cardiology issued by the AMB/SBC.
- During the formation period, the trainee must fulfill the full course load established by the theoretical-practical program.

#### 3.1.5. The Theoretical-Practical Program

- The minimum training period is 24 consecutive months, with 30 days of vacation per year, scientific improvement, and participation in congresses and meetings related to the specialization.
- The training program must provide trainees with a complete formation, with mastery of the techniques and knowledge related to cardiovascular interventions. The first year should focus on fundamental training in radiation protection, vascular accesses, diagnostic percutaneous procedures, low complexity coronary interventions, and their complications. The second year should include and focus on training in high complexity coronary interventions, approaches to structural heart disease, extracardiac vascular procedures, and their complications.
- The direct participation of the trainee in diagnostic and therapeutic cardiovascular procedures should always take place under preceptor supervision, and all pertinent activities should be duly registered in the current SBHCI database.
- Throughout the duration of the training period, the trainee must act as first operator, under supervision, in at least:
  - 400 diagnostic cardiovascular procedures.
  - 200 PCI.
  The minimum training syllabus in hemodynamics and interventional cardiology should comprehend:^3-7^
- A review of historical aspects relevant to hemodynamics and interventional cardiology.
- Basic concepts of ionizing radiation, image formation, and radiation protection (Annex 1).
- Vascular accesses (vascular anatomy; choice and techniques for arterial and venous access in multiple sites [radial/ulnar, femoral, jugular, transapical, transhepatic, carotid, subclavian/axillary, cavo-aortic]; techniques for obtaining hemostasis; and treatment of vascular complications).
- Ultrasound imaging for obtaining vascular access.
- Vascular hemostasis devices (indications, benefits, limitations, and complications).
- Manometry recordings (critical evaluation of recording quality and its functioning; and analysis of arterial and venous pressure curves in different cardiac cavities and vascular circuits under normal conditions and in pathological situations, including assessment of ventricular diastolic function).
- Determination of cardiac output by the Fick principle and the thermodilution method.
- Calculation of valve areas, vascular resistances, and arteriovenous shunts.
- Evaluation of hemodynamic response to pharmacological agents to study ventricular performance and pulmonary vascular reactivity.
- Means of contrast (types, doses, complications, prevention and treatment of adverse reactions).
- Knowledge of cardiac, coronary, and vascular radiological anatomy and the corresponding angiographic projections for proper performance of cardiovascular procedures.
- A review of cardiovascular anatomy and physiology with a focus on interventional cardiology.
- Cardiovascular pathology and physiopathology (determinants of atherosclerosis and thrombosis; systemic manifestations of atherosclerosis and risk factors that contribute to its development; established guidelines for modifying these risk factors; physiopathology, clinical manifestations, natural history, assessment, and management of other structural and functional cardiovascular diseases; prothrombotic states, encompassing hereditary and acquired disorders; and proinflammatory states that contribute to appearance of unstable coronary lesions).
- Indications and contraindications for therapeutic and diagnostic percutaneous cardiovascular procedures.
- Interpretation of images and quantitative angiography for assessment of coronary lesions, valve dysfunctions, and systolic ventricular function.
- Methods for evaluating functional significance of coronary obstructions.
- Methods for obtaining and interpreting intravascular imaging.
- Technical knowledge of materials utilized for diagnostic and interventional cardiovascular procedures.
- Recognition and management of complications of diagnostic and therapeutic cardiovascular procedures.
- Pharmacology applied to diagnostic and therapeutic percutaneous cardiovascular procedures.
- Hemodynamic and angiographic diagnoses of the main congenital and structural cardiovascular diseases in children and adults.
- Indications, contraindications, techniques, and limitations of diverse therapeutic procedures in interventional cardiology for congenital heart diseases (atrial septostomy [diverse techniques] and pulmonary and aortic valvuloplasty; angioplasty and pulmonary artery stent implantation, conduct and other arteries and veins; aortoplasty and aortic stent; occlusion techniques, embolizations and cardiac occlusion devices; and transcatheter pulmonary valve implantation [TPVI]).
- Peculiar technical aspects of all percutaneous cardiovascular intervention devices.
- Indications, contraindications, methods, techniques, and limitations of diverse therapeutic procedures in interventional cardiology (coronary and vascular interventions; utilization of distal protection devices; valvuloplasties; alcohol septal ablation in obstructive hypertrophic cardiomyopathy; embolization of coronary arteries to treat complications and other vascular beds for therapeutic purposes; and retrieval of intravascular foreign bodies by percutaneous methods).
- Indications, contraindications, techniques, and limitations of diverse percutaneous procedures employed to treat structural heart diseases, such as percutaneous aortic valve implantation (transcatheter aortic valve replacement (TAVR), transcatheter mitral valve repair (TMVR), atrial appendage occlusion, percutaneous treatment of paravalvular leaks and patent foramen ovale (PFO) closure.
- Indications and management of ventricular assist devices.
- Critical analysis of published studies, in accordance with the principles of medicine based on scientific evidence.
- Ethics and compliance in interventional cardiology.
- Writing and obtaining consent forms in interventional cardiology. Examples of consent forms suggested by the SBHCI for the most common procedures may be found in Annexes 2 to 15.
- Writing reports in interventional cardiology (Annex 16).

## 4. Approval, Maintenance, and Revalidation of Accredited Centers

The definition of norms for accreditation and maintenance of hemodynamics and interventional cardiology training centers will be governed by self-regulation of which the Corporate Advisory Council is in charge. Verification of compliance with the requirements established in this Position Paper is the responsibility of the SBHCI’s Permanent Certification Commission (Comissão Permanente de Certificação - CPC). To this end, a Verification Committee will be designated, consisting of three members, one belonging to the Directory Board, one belonging to the Advisory Council, and one belonging to the CPC.

Training centers will be submitted to a re-registration process every four years. In the event that the requirements of these guidelines are infringed, the Verification Committee will advise the coordinator of the training center to redress the irregularities observed, by means of a report based on the corrections necessary for the training center’s maintenance. Six months later, a new inspection will be carried out. In the event that the recommendations have not been met, a final opinion will be emitted by the Advisory Council, deciding on the center’s loss of accreditation.

Loss of accreditation of a training center will also occur under any one of the following conditions:

- Absence of at least one trainee who has completed training within a continuous four-year period.
- Absence of at least one graduate trainee from the training center who has passed the test to obtain certification in the area of interventional cardiology and interventional cardiology from the SBHCI, over the past four years.

Formal authorization is mandatory from the technical director of the hospital where the training center will operate.

## 5. Annual Evaluation of Trainees and Training Centers

At the end of each year of training, the CPC, in conjunction with the SBHCI, will make a structured form (Annex 17) available on the SBHCI intranet for all trainees, who will confidentially assess whether the training center is fulfilling this Position Paper’s recommendations. Each form must be forwarded to the SBHCI.

## 6. Percutaneous Intervention in Congenital and Structural Heart Diseases and in the Extracardiac Vascular Bed

As observed, the theoretical-practical program which is applied over the two-year period is rich and comprehensive, and it allows interventional cardiologists, following training and certification, to act with proficiency in the treatment of coronary artery disease in their most varied forms of presentation. Furthermore, during this formative period or even after the conclusion of this phase, it is possible to participate in specific training processes, which will provide interventional cardiologists with the competences necessary to act in congenital and structural heart diseases and in the extracardiac arterial and venous vascular bed. We will subsequently detail the specific processes that ensure that an interventional cardiologist has obtained competences in the areas mentioned.

### 6.1. Congenital Heart Diseases

The knowledge, the abilities, and the training necessary for therapeutic interventions of congenital heart disease in children and adults are different than those required for PCI. Knowledge of the natural history of diverse congenital heart diseases is a prerequisite for the procedure’s safety and effectiveness. Trainees are also required to know the indications for percutaneous treatment, as well as diverse types of surgery (palliative and corrective), hemodynamic evaluation and interpretation of diverse conditions in the catheterization laboratory, study and management of pulmonary hypertension, angiography, and interpretation of reports and complementary exam images, such as transthoracic echocardiography (TTE), transesophageal echocardiography (TEE), intracardiac echocardiography, CT, and MR. In contemporary interventional cardiology practice, we also recommend that trainees develop abilities for software or computer programs dedicated to reconstruction and measurement of cardiovascular structures. This new set of abilities aims to increase the capacity for planning and carrying out highly complex procedures, with the goal of increasing efficacy and safety for patients.

Training should proceed starting with less complex procedures until the abilities have been acquired which will allow trainees to perform procedures considered more complex. Among the abilities that should be acquired, basic training, by means of supervised practice, includes handling diverse vascular accesses, guides, wires, balloons, and devices, and, especially, orientation on how to anticipate, recognize, and treat possible complications. Great effort should be put forth to disseminate precautions with ionizing radiation and rational use of different means of contrast. Concomitant to this technical formation, it is recommended that trainees participate in didactic sessions, case discussions, post-procedure follow-up of patients, outpatient clinical follow-up, research, databanks and registries, assessment of quality and results, and participation in multidisciplinary groups (heart teams). Also recommended are training and familiarity with the particularities of handling newborns and young infants.

We consider that, for a complete formation, this training should last at least two years for pediatric or adult cardiologists or at least one additional year for general interventional cardiologists. Due to the numerous procedures currently performed for congenital and structural heart disease, the minimum number of exams carried out in order to have proper qualification varies in accordance with the complexity of the condition and intervention; however, qualified centers for formation of interventional cardiologists in congenital heart diseases should perform at least 100 therapeutic procedures per year, and trainees should participate as first operators in at least 40 cases during the training period.

Subsequently, we will describe the recommendations for acquisition and maintenance of competences in interventional treatment of congenital heart diseases, which are here divided by degree of complexity.

#### 6.1.1 Basic Interventions in Congenital Heart Diseases

##### 6.1.1.1 Interatrial Communication Occlusion

###### 6.1.1.1.1. Basic Knowledge

- Natural history, classification, and hemodynamic repercussion of septal atrial defects.
- Indications for intervention.
- Proper differentiation between simple and complex defects.

###### 6.1.1.1.2. Interventional Cardiologists’ Abilities

- Knowledge and proper handling of introducers, sheaths, guidewires, and catheters utilized.
- Interpretation and familiarity with different imaging tests: atrial septal characteristics; determination of defect location, number, borders, and adjacent structures by TTE, TEE, intracardiac echocardiography, and fluoroscopy.
- Invasive assessment of pulmonary vascular pressure and reactivity, including the need for temporary balloon occlusion.
- When and how to use balloons for measurement.
- In-depth knowledge of different available devices, their characteristics, sizes, and forms of release.
- Mastery of techniques necessary to remove devices in the event of device embolization.
- Patient care and recognition of immediate and late complications and long-term patient guidance.

##### 6.1.1.2. Ductus Arteriosus Occlusion

###### 6.1.1.2.1. Basic Knowledge

- Natural history, classification, and hemodynamic repercussions of patent ductus arteriosus (PDA).
- Knowledge of the different anatomical types of PDA.
- Proper differentiation of simple or complex PDA, associated congenital anomalies, and pulmonary hypertension.
- Indications or contraindications for intervention.

###### 6.1.1.2.2. Interventional Cardiologists’ Abilities

- Evaluation of adequate access route for patients.
- Knowledge and proper handling of introducers, sheaths, guidewires, and catheters utilized.
- Invasive assessment of pulmonary vascular pressure and reactivity, including the need for temporary balloon occlusion.
- Evaluation of characteristics of the ductus arteriosus by means of diverse imaging methods: TTE, CT, MR, and angiography.
- Techniques for crossing the ductus: anterograde and retrograde.
- In-depth knowledge of different available devices, their characteristics, sizes, and forms of release.
- Mastery of techniques necessary to remove devices in the event of device embolization.
- Patient care and recognition of immediate and late complications and long-term patient guidance.

##### 6.1.1.3. Pulmonary Valvuloplasty

###### 6.1.1.3.1. Basic Knowledge

- Natural history, classification and hemodynamic repercussion of pulmonary stenosis.
- Knowledge of different types and etiology of pulmonary stenosis, as well as associated anomalies.
- Indications or contraindications for intervention.

###### 6.1.1.3.2. Interventional Cardiologists’ Abilities

- Knowledge and proper handling of introducers, sheaths, guidewires, and catheters utilized.
- Hemodynamic interpretation of pulmonary stenosis in the catheterization laboratory.
- Assessment of characteristics of pulmonary stenosis by means of diverse imaging methods: TTE, CT, MR, and angiography.
- Techniques for crossing pulmonary stenosis.
- Knowledge of the different balloons available for the procedure.
- Patient care and recognition of immediate and late complications and long-term patient guidance.

##### 6.1.1.4. Aortic Valvuloplasty

###### 6.1.1.4.1. Basic knowledge

- Natural history, classification, and hemodynamic repercussion of congenital aortic stenosis (AoS).
- Knowledge of different types of AoS, as well as associated anomalies.
- Indications or contraindications for intervention.

###### 6.1.1.4.2. Interventional Cardiologists’ Abilities

- Assessment of vascular access: carotid, axillary, or femoral; puncture or dissection.
- Knowledge and proper handling of introducers, sheaths, guidewires, and catheters utilized.
- Hemodynamic interpretation of AoS in the intervention laboratory.
- Assessment of characteristics of AoS by means of diverse imaging methods: TTE, TEE, CT, MR, and angiography.
- Techniques for crossing the AoS.
- Knowledge of different balloons available for this procedure and their choice in relation to ring size.
- Stimulation of very high frequencies by means of a pacemaker (rapid pacing).
- Patient care and recognition of immediate and late complications and long-term patient guidance.

#### 6.1.2. Complex Interventions in Congenital Heart Diseases

##### 6.1.2.1. Interventricular Communication Occlusion

###### 6.1.2.1.1. Basic Knowledge

- Natural history, localization, characteristics, and associated defects. Simple or complex interventricular communication (IVC).
- Hemodynamic repercussion and assessment of pulmonary hypertension.
- Feasibility and indications for intervention.
- Therapeutic and prognostic options. Risk of total atrioventricular block (TAB).

###### 6.1.2.1.2. Interventional Cardiologists’ Abilities

- Knowledge and proper handling of introducers, sheaths, guidewires, and catheters utilized.
- Interpretation and familiarity with different imaging exams: characteristics of the ventricular septum, determination of defect location, number, borders, and adjacent structures by TTE, TEE, CT, MR, and angiography.
- Choice of access route (superior vena cava, inferior vena cava, transapical, etc.).
- Invasive assessment of pulmonary vascular pressure and reactivity.
- Appropriate angiography for characterization of the defect.
- Techniques for crossing and releasing the device: anterograde and retrograde.
- Proper technical knowledge of different devices available, characteristics, sizes, and forms of release.
- Mastery of techniques necessary to remove devices in the event of embolization.
- Patient care and recognition of immediate and late complications and long-term patient guidance.

##### 6.1.2.2. Angioplasty and Stent Implantation for Coarctation of the Aorta

###### 6.1.2.2.1. Basic Knowledge

- Natural history and treatment of native coarctation of the aorta (CoA) or post-operative recoarctation.
- Knowledge of different types and etiology of simple or complex CoA and associated anomalies.
- Assessment of characteristics of CoA by means of diverse imaging methods: TTE, TEE, CT, MR, and angiography.
- Indications for intervention.

###### 6.1.2.2.2. Interventional Cardiologists’ Abilities

- Knowledge and proper handling of sheaths, guidewires, and catheters.
- Choice of adequate access route: femoral, carotid, radial, axillary; puncture or dissection.
- Hemodynamic and angiographic interpretation of CoA in the intervention laboratory.
- Techniques for crossing the coarctation: anterograde or retrograde.
- Knowledge and appropriate choice of different balloons and covered or uncovered stents available for this procedure and their techniques.
- Recognition and treatment of acute complications.
- Knowledge and techniques for utilizing different hemostasis devices.
- Long-term care.

##### 6.1.2.3. Angioplasty and Stent Implantation for Pulmonary Artery Stenosis

###### 6.1.2.3.1. Basic Knowledge

- Natural history and knowledge of etiology, whether congenital or acquired, isolated or multiple, proximal or distal, and associated malformations.
- Appropriate assessment of pulmonary arteries by means of diverse imaging methods: TTE, TEE, CT, MR, angiography, and scintigraphy.
- Indications for intervention.

###### 6.1.2.3.2. Interventional Cardiologists’ Abilities

- Knowledge and proper handling of sheaths, guidewires, and catheters utilized.
- Choice of adequate vascular access.
- Hemodynamic and angiographic assessment of pulmonary stenoses.
- Knowledge and appropriate choice of different balloons, stents, and covered stents available for this procedure and their techniques.
- Recognition and treatment of acute complications.
- Medium and long-term care and orientations for the patient.

##### 6.1.2.4. Angioplasty and Stent Implantation for Pulmonary Vein Stenosis

###### 6.1.2.4.1. Basic Knowledge

- Natural history and knowledge of etiology, whether congenital or post-operative.
- Appropriate assessment of pulmonary veins by means of diverse imaging methods: TTE, TEE, CT, MR, angiography, and scintigraphy.
- Indications for intervention.

###### 6.1.2.4.2. Interventional Cardiologists’ Abilities

- Knowledge and proper handling of sheaths, guidewires, and catheters utilized.
- Choice of adequate access route.
- Transseptal puncture: techniques (guided by fluoroscopy and echocardiography) and complications.
- Selective hemodynamic and angiographic assessment of pulmonary veins.
- Knowledge and appropriate choice of different balloons and stents available for this procedure and their techniques.
- Recognition and treatment of acute complications.
- Medium and long-term care.

##### 6.1.2.5. Angioplasty and Stent Implantation in Surgical Conduits, Tunnels, and Homografts

###### 6.1.2.5.1. Basic Knowledge

- Natural history of the different types of material utilized: biological or synthetic, valved or not valved.
- Knowledge of anatomical and physiological differences in intra- or extracardiac surgical conduits and tunnels.
- Appropriate understanding of the anatomy and hemodynamics of surgical procedures performed in complex heart diseases, univentricular physiology, transposition of great arteries, corrected transposition of great arteries; repercussions of ventricle pressure and volume overload.
- Appropriate assessment of anatomy and physiology by means of diverse imaging methods: echocardiography, CT, MR, angiography, and scintigraphy.
- Indications for intervention.

###### 6.1.2.5.2. Interventional Cardiologists’ Abilities

- Knowledge and proper handling of sheaths, guidewires, and catheters utilized.
- Choice of vascular access.
- Hemodynamic and angiographic assessment of surgical conduits and tunnels.
- Knowledge and appropriate choice of different balloons, stents, and covered stents available for this procedure and their techniques.
- Recognition and treatment of acute complications.
- Medium and long-term care and orientations for the patient.

### 6.2. Structural Heart Diseases

Structural heart diseases encompasses congenital and acquired conditions that involve major cardiovascular structures, excluding coronary atherosclerotic and peripheral vascular diseases. Formal training in structural and congenital heart diseases in adults takes place during the specialization phase, even in developed countries.^8^ With the advent of percutaneous interventions for the treatment of structural defects and valve diseases, such as TAVR, TPVI, and TMVR, as well as the expansion of occlusion procedures with intra- and extracardiac shunts, among others, it is clear that there is a need to create basic requirements for training interventional cardiologists who are interested in performing these procedures.

As the complexity of the conditions increases, the level of training goes from a “basic” stage to more “advanced” levels. Training for occlusion of atrial septal defects requires less advanced abilities and tools than those that are required for mitral paravalvular leak closure, for instance. Training in formation centers should seek to hierarchize this process. In the same manner, for interventional cardiologists who are already acting in the field, the tutorial process, with the figure of an instructor or proctor, is fundamental to determining which steps to go through, from less complex to more complex scenarios.

#### 6.2.1. Basic Interventions in Structural Cardiovascular Diseases

##### 6.2.1.1. Catheterization of Left Chambers following Transseptal Puncture

###### 6.2.1.1.1. Basic Knowledge

- Normal anatomy and morphospatial variations resulting from diverse conditions (right and/or left atrial dilatation, ascending aortic dilatation, dextrocardia, heterotaxy, etc.).
- Hemodynamic interpretation of pressure curves.
- Appropriate assessment and recognition of atrial septal structures by means of diverse imaging methods: TTE, TEE, intracardiac echocardiography, and fluoroscopic markers.
- Indications for intervention.

###### 6.2.1.1.2. Interventional Cardiologists’ Abilities

- Percutaneous access for transseptal puncture.
- Transseptal introducers, wires, needles, and other devices, such as radiofrequency.
- Selective puncture guided by TEE.

##### 6.2.1.2. Aortic Valvuloplasty in Adults

###### 6.2.1.2.1. Basic Knowledge

- Natural history and etiology of AoS.
- Hemodynamics of severe AoS with high and low gradients.
- Interpretation and familiarity with different imaging exams of the aortic valve: TTE, TEE, CT, MR, angiography.
- Knowledge of current guidelines for treatment of AoS.
- Therapeutic options and outcomes.
- Indications for intervention.

###### 6.2.1.2.2. Interventional Cardiologists’ Abilities

- Hemodynamic interpretation of AoS.
- Access choice.
- Techniques for crossing the stenotic aortic valve.
- Sheaths, wires, and catheters utilized.
- Balloon catheters for valvuloplasty.
- Stimulation of very high frequencies by means of a pacemaker (rapid pacing).
- Familiarity in managing devices for vascular suture.
- Recognition and rapid management of complications (vascular occlusions, dissections, thromboembolism, hemodynamic collapse, retroperitoneal bleeding, cardiac perforations, arrhythmias/atrioventricular blocks, coronary occlusion, etc).
- Immediate and long-term post-procedural care.

##### 6.2.1.3. Patent Foramen Ovale Occlusion

PFO is observed in approximately 25% of the general population, and it may thus coexist, by chance, in patients with strokes or thromboembolic phenomena that are undefined in nature.^9^ Epidemiological data, however, have established a clear relation between PFO and stroke of undetermined origin.^10-14^ Additionally, studies have documented higher rates of systemic embolization among patients who have venous thrombosis or debris and concomitant PFO.^15-18^ Many reports have also demonstrated direct evidence of thrombi adherent to the PFO.^19,20^ Finally, and most importantly, randomized studies have demonstrated that PFO occlusion significantly reduces stroke recurrence in comparison with isolated medical therapy.^21-24^

Based on a document signed by several European scientific societies, PFO occlusion or closure in the scenario of stroke or paradoxical systemic embolic events of undefined causes (related to PFO) should be performed in select patients, between the ages of 18 and 65, as a form of secondary prevention.^25^ Within this same scenario, it has also been demonstrated that the cost-effectiveness relationship is favorable for PFO occlusion, in comparison with isolated medical treatment.^26^

###### 6.2.1.3.1. Basic Knowledge

- Natural history and mechanisms of paradoxical thromboembolic events; stroke related to PFO; hemodynamic repercussion of atrial septal defects.
- Medical management of stroke related to PFO.
- Interpretation and familiarity with different imaging exams related to the atrial septum, adjacent structures, and the brain structures as well: TTE, TEE, intracardiac echocardiography, MR, CT, transcranial Doppler, and fluoroscopy.
- Knowledge of current guidelines for PFO occlusion in the scenario of paradoxical embolism (secondary prevention).
- Indications for intervention.

###### 6.2.1.3.2. Interventional Cardiologists’ Abilities

- Techniques for crossing the PFO.
- Knowledge and proper handling of introducers, sheaths, guidewires, and catheters utilized.
- Knowledge and abilities with imaging methods for guiding the procedure: TEE, intracardiac echocardiography, and fluoroscopy.
- When and how to use balloons for measurement.
- In-depth knowledge of different devices available, their characteristics, sizes, and forms of release.
- Mastery of techniques necessary to remove devices in the event of embolization.
- Recognition and rapid management of complications (vascular occlusion, dissection, thromboembolism, hemodynamic collapse, cardiac perforation, arrhythmias/atrioventricular block, coronary occlusion, etc.).
- Immediate and long-term post-procedural care.

#### 6.2.2. Complex Interventions in Structural Cardiovascular Diseases

##### 6.2.2.1. Transapical Ventricular Access

###### 6.2.2.1.1. Basic Knowledge

- Normal anatomy and morphospatial variations resulting from diverse conditions (right and/or left atrial dilatation, ascending aortic dilatation, dextrocardia, heterotaxy, etc.).
- Hemodynamic interpretation of pressure curves.
- Familiarity with different imaging exams (CT, TTE, TEE, intracardiac echocardiography).
- Indications for intervention.

###### 6.2.2.1.2. Interventional Cardiologists’ Abilities

- Micropuncture needles, wires, and introducers.
- Selective puncture guided by CT, ultrasound, and fluoroscopy.
- Knowledge and techniques for utilization of different hemostasis devices.
- Immediate and long-term post-procedural care.

##### 6.2.2.2. Transhepatic Access

###### 6.2.2.2.1. Basic Knowledge

- Normal anatomy and morphospatial variations resulting from diverse conditions.
- Indications for intervention.

###### 6.2.2.2.2. Interventional Cardiologists’ Abilities

- Micropuncture needles, wires, and introducers.
- Selective puncture guided by ultrasound and fluoroscopy.
- Knowledge and techniques for utilization of different hemostasis devices
- Immediate and long-term post-procedural care.

##### 6.2.2.3. Mitral and Tricuspid Valvuloplasty in Adults

###### 6.2.2.3.1. Basic Knowledge

- Natural history and etiology of mitral and tricuspid stenosis.
- Hemodynamics of severe mitral and tricuspid stenoses.
- Interpretation and familiarity with different imaging methods related to the aortic and tricuspid valves: echocardiography, CT, MR, angiography.
- Knowledge of current guidelines for treatment of mitral and tricuspid stenoses.
- Therapeutic options and outcomes.
- Indications for intervention.

###### 6.2.2.3.2. Interventional Cardiologists’ Abilities

- Hemodynamic interpretation of pressure curves in mitral and tricuspid stenoses in the hemodynamics laboratory.
- Choice of vascular access.
- Selective transseptal puncture (for mitral stenosis).
- Techniques for crossing mitral and tricuspid stenoses.
- Sheaths, wires, and catheters utilized.
- Balloon catheters for valvuloplasty.
- Knowledge and techniques for utilization of different hemostasis devices.
- Recognition and rapid management of complications (vascular occlusions, dissections, thromboembolism, hemodynamic collapse, retroperitoneal bleeding, cardiac perforations, tamponade, arrhythmias/atrioventricular blocks, etc.).
- Immediate and long-term post-procedural care.

##### 6.2.2.4. Balloon Pericardiotomy and Pericardiocentesis

###### 6.2.2.4.1. Basic Knowledge

- Natural history of recurrent or malign pericardial effusion.
- Therapeutic options and outcomes.
- Pericardial sac anatomy.
- Indications for intervention.

###### 6.2.2.4.2. Interventional Cardiologists’ Abilities

- Techniques for percutaneous access to the pericardial sac.
- Puncture guided by echocardiography and fluoroscopy.
- Needles, wires, introducers, catheters, and balloon catheters.
- Immediate and long-term post-procedural care.

##### 6.2.2.5. Post-infarction Interventricular Communication Occlusion

###### 6.2.2.5.1. Basic Knowledge

- Natural history of post-infarction IVC (anterior and inferior infarctions).
- Management of cardiogenic shock.
- Hemodynamic repercussion and assessment of pulmonary hypertension.
- Interpretation and familiarity with different imaging exams related to characteristics of the ventricular septum, determination of defect location, number, and adjacent structures: TTE, TEE, CT, MR, and angiography.
- Feasibility and indications for intervention.

###### 6.2.2.5.2. Interventional Cardiologists’ Abilities

- Hemodynamic interpretation of post-infarction IVC in the hemodynamics laboratory.
- Choice of vascular access (superior vena cava, inferior vena cava, transapical, etc.).
- Techniques for crossing the IVC.
- Knowledge and proper handling of sheaths, guidewires, and catheters utilized.
- Knowledge of when and how to use balloons for measurement.
- Appropriate angiography for defect characterization.
- Techniques for crossing and releasing the device: anterograde and retrograde.
- Proper technical knowledge of different devices available, characteristics, sizes, and forms of release.
- Mastery of techniques necessary to remove devices in the event of embolization.
- Recognition and rapid management of complications (vascular occlusions, dissections, thromboembolism, hemodynamic collapse, cardiac perforations, cardiac tamponade, device embolism, arrhythmias/atrioventricular blocks, coronary occlusion etc.).
- Immediate and long-term post-procedural care.

##### 6.2.2.6. Occlusion of Paravalvular Leaks

###### 6.2.2.6.1. Basic Knowledge

- Natural history of paravalvular leaks.
- Paravalvular leaks in mechanical and biological valve prostheses.
- Recognition and clinical management of mechanical device hemolysis.
- Interpretation and familiarity with different imaging exams related to accurate localization of the leak: TTE, TEE, CT, MR, and angiography.
- Indications for intervention.

###### 6.2.2.6.2. Interventional Cardiologists’ Abilities

- Hemodynamic interpretation of paravalvular leaks in the hemodynamics laboratory.
- Choice of vascular access: retrograde, anterograde transseptal, and transapical.
- Selective transapical puncture.
- Techniques for crossing paravalvular leaks.
- Knowledge and proper handling of introducers, sheaths, guidewires, and catheters utilized.
- Proper technical knowledge of different devices available, their characteristics, sizes, and forms of release.
- Mastery of techniques necessary to remove devices in the event of embolization.
- Recognition and rapid management of complications (vascular occlusion, dissection, thromboembolism, hemodynamic collapse, cardiac perforation, cardiac tamponade, device embolism, arrhythmias/atrioventricular block, coronary occlusion etc.).
- Immediate and long-term post-procedural care.

##### 6.2.2.7. Left Ventricular Pseudoaneurysm Occlusion

###### 6.2.2.7.1. Basic Knowledge

- Natural history of ventricular pseudoaneurysms.
- Pseudoaneurysms as complication of mechanical and biological valve prostheses.
- Recognition and clinical management of mechanical device hemolysis.
- Management of cardiac insufficiency.
- Interpretation and familiarity with different imaging exams: accurate localization and characterization of the pseudoaneurysm by TTE, TEE, CT, MR, and angiography.
- Indications for intervention.

###### 6.2.2.7.2. Interventional Cardiologists’ Abilities

- Choice of access route: retrograde, anterograde transseptal, and transapical.
- Selective transseptal puncture.
- Selective transapical puncture.
- Techniques for entering the left ventricular pseudoaneurysm.
- Knowledge and proper handling of introducers, sheaths, guidewires, and catheters utilized.
- Proper technical knowledge of different devices available, their characteristics, sizes, and forms of release.
- Mastery of techniques necessary to remove devices in the event of embolization.
- Recognition and rapid management of complications (vascular occlusion, dissection, thromboembolism, hemodynamic collapse, cardiac perforation, cardiac tamponade, device embolism, arrhythmias/atrioventricular block, coronary occlusion etc.).
- Immediate and long-term post-procedural care.

##### 6.2.2.8. Occlusion of Endovascular Endoleaks

###### 6.2.2.8.1. Basic Knowledge

- Natural history and recognition of endovascular endoleaks.
- Endovascular endoleaks in different endoprosthesis types.
- Knowledge of the collateral and arterial ramifications of the aorta.
- Interpretation and familiarity with different imaging exams: CT, MR, angiography, and ultrasound.
- Indications for intervention.

###### 6.2.2.8.2. Interventional Cardiologists’ Abilities

- Choice of vascular access.
- Direct selective access.
- Knowledge and proper handling of introducers, sheaths, guidewires, and catheters utilized.
- Proper technical knowledge of different types of occlusion devices and chemical agents.
- Mastery of techniques necessary to remove devices in the event of embolization.
- Recognition and rapid management of complications (vascular occlusions, dissections, thromboembolism, hemodynamic collapse, cardiac perforations, cardiac tamponade, device embolism, arrhythmias/atrioventricular blocks, coronary occlusion, etc.).
- Immediate and long-term post-procedural care.

##### 6.2.2.9. Aortic Pseudoaneurysm Occlusion

###### 6.2.2.9.1. Basic Knowledge

- Natural history and etiology of aortic pseudoaneurysms.
- Interpretation and familiarity with different imaging exams related to accurate localization and characterization of the pseudoaneurysm: ultrasound, CT, MR, and angiography.
- Indications for intervention.

###### 6.2.2.9.2. Interventional Cardiologists’ Abilities

- Choice of vascular access.
- Knowledge and proper handling of introducers, sheaths, guidewires, and catheters utilized.
- Proper technical knowledge of different types of occlusion devices, chemical agents, covered stents, and endoprostheses.
- Mastery of techniques necessary to remove devices in the event of device embolization.
- Recognition and rapid management of complications (vascular occlusion, dissection, thromboembolism, hemodynamic collapse, cardiac perforation, cardiac tamponade, device embolism, arrhythmias/atrioventricular block, coronary occlusion etc.).
- Immediate and long-term post-procedural care.

##### 6.2.2.10. Hypertrophic Cardiomyopathy and Alcohol Septal Ablation

Hypertrophic cardiomyopathy is the most common genetic cardiovascular disease, with an estimated prevalence of 0.2% in the general population.^27^ Interventional cardiologists who perform this procedure should possess extensive knowledge of the results, limitations, and complications of medical therapy, surgical myectomy, dual-chamber pacemaker pacing, and alcohol septal ablation itself.^27-29^

Alcohol septal ablation should involve a multidisciplinary program that includes the contributions of a cardiac surgeon, an echocardiographist, a clinical cardiologist, and an electrophysiologist. The first procedures should be supervised by an experienced operator in training centers or in services outside of training centers under the supervision of a medical instructor (proctor).

###### 6.2.2.10.1. Basic Knowledge

- Natural history and etiologies of left ventricular outlet obstructions.
- Hemodynamics of left ventricular outlet obstructions.
- Interpretation and familiarity with different imaging exams: TTE, TEE, CT, MR, and angiography.
- Knowledge of current guidelines for treatment of hypertrophic cardiomyopathy.
- Therapeutic options.
- Indications for intervention.

###### 6.2.2.10.2. Interventional Cardiologists’ Abilities

- Hemodynamic interpretation of left ventricular outlet obstruction.
- Choice of vascular access.
- Sheaths, wires, balloons, and catheters utilized.
- Angiographic projections for septal ablation.
- Pacemaker in the right ventricle during intervention.
- Ablative substances (alcohol, microspheres, etc.).
- Recognition and rapid management of complications (vascular occlusion, dissection, thromboembolism, hemodynamic collapse, extension of infarction, iatrogenic post-infarction IVC, cardiac perforation, arrhythmias/atrioventricular block, coronary occlusion, etc.).
- Immediate and long-term post-procedural care.

##### 6.2.2.11. Left Atrial Appendage Occlusion

Oral anticoagulation is indicated as a class I recommendation, level of evidence A, for prevention of thromboembolic stroke in patients with non-valvular atrial fibrillation (NVAF) and CHA_2_DS_2_-VASc scores ≥ 2, in both Brazilian^30^ and international^31,32^ guidelines. There are, however, several absolute and relative contraindications to the use of this therapy, either with the use of vitamin K antagonists or direct action oral anticoagulants^33^ (Chart 3).

**Chart 3:** Absolute and relative contraindications to the use of oral anticoagulation

• Previous significant bleeding
• Previous intracranial bleeding
• Symptomatic bleeding in a critical organ (e.g., ocular, pericardial, medullary)
• High risk of bleeding
• Frailty/frequent falls
• Comorbidities (e.g., intestinal angiodysplasia, renal failure, blood dyscrasia)
• Lack of adherence to treatment
• Labile INR
• Anticoagulant intolerance
• Use of double antiplatelet therapy
• Refusal to use the medication
• Occupational risk

INR: international normalized ratio.

Based on the rationale that more than 90% of intracardiac thrombi that are formed as a result of NVAF are localized in the left atrial appendage (LAA),^34^ percutaneous left atrial appendage occlusion (LAAO) has proven to be a non-inferior alternative, in relation to the occurrence of thromboembolism, and it is superior in terms of late mortality, when compared to oral anticoagulation with warfarin.^35,36^ The II Brazilian Guidelines for Atrial Fibrillation recommend LAAO for patients with high risks of thromboembolic phenomena and contraindications to the use of oral anticoagulants (class IIa, level of evidence B), and for patients sustaining a ischemic stroke of cardioembolic origin occurring in the presence of adequate oral anticoagulant use (class IIa, level of evidence C).^30^

Notwithstanding its growing utilization, LAAO is not yet an intuitive procedure for interventional surgeons, which is reflected by the slower and more gradual learning curve and by the potential associated complications. In addition to technical abilities for the intervention itself, LAAO requires proficiency in several stages of management for these patients who are generally elderly and complex, including proper indication for the procedure, interpretation of cardiac angiography and pre- and trans-operative echocardiography imaging, and management of specific protocols of medication and late follow-up. In this manner, with the aim of obtaining better safety and effectiveness in the intervention, and in accordance with international propositions,^36-38^ these guidelines recommend the establishment of prerequisites for institutions and professionals who wish to dedicate themselves to LAAO, in addition to a consistent model for acquiring competence in this intervention.

###### 6.2.2.11.1. Institutional Prerequisites

The institution should have an established service for structural or congenital heart diseases and/or electrophysiology equipped with an infrastructure that includes, among other things, a hybrid room or a cardiac catheterization laboratory with fixed hemodynamic equipment; it is considered not adequate performing these procedures with a C-arm.

There should be a local echocardiography service, with a capacity for performing transthoracic and transesophageal exams with an experienced operator. Anesthesiologists experienced in complex cardiovascular interventions should be part of the local team. The institution should also have a structured cardiac surgery service. It is not considered necessary to keep a cardiac surgery team on stand-by during the procedure; it should, nonetheless, be possible to activate this team rapidly, if necessary.

###### 6.2.2.11.2. Basic Knowledge

- Basic knowledge about management of patients with atrial fibrillation, including mastery of tools for assessing risk of stroke and bleeding.
- Detailed understanding of cardiac anatomy, surrounding structures, and the anatomical variability of the LAA, with the ability to interpret invasive pressure curves, fluoroscopy, echocardiography, and cardiac angiotomography images related to the procedure and its possible complications.
- Interpretation and familiarity with different imaging exams related to the LAA: TTE, TEE, CT, MR, and angiography.
- Knowledge of current guidelines for LAAO.
- Therapeutic options and outcomes.
- Indications for intervention.

###### 6.2.2.11.3. Interventional Cardiologists’ Abilities

- Interpretation of LAA images.
- Selective transseptal puncture.
- Safe access to the LAA.
- Sheaths, wires, and catheters utilized.
- Proper technical knowledge of the different devices available, their characteristics, sizes, forms of release, and contraindications.
- Recognition and rapid management of complications (vascular occlusions, dissections, thromboembolism, hemodynamic collapse, cardiac perforations, cardiac tamponade, device embolism, arrhythmias/atrioventricular blocks, coronary occlusion etc.).
- Immediate and long-term post-procedural care.

###### 6.2.2.11.4. Acquisition of Competence and Training Models

Once these prerequisites have been met, the acquisition of competence for an operator to become independent in this intervention should follow a structured, consistent model. Training should involve practical activities that include simulation of cases of transseptal puncture and implantation of LAA occluder prostheses, with manipulation of the material utilized during the procedure. During the first effective implants, the operator should be assisted by a medical instructor (proctor) with proven experience in the intervention.

The duration of the learning curve for LAAO varies significantly, in accordance with the operator’s degree of familiarity with congenital and/or structural heart disease procedures and the frequency with which the procedure is performed. There is no consensus in the literature with respect to the minimum number of cases required in order to complete this learning curve;^39,40^ nevertheless, within the Brazilian context, considering the complexity of the intervention, the practical experience of the authors of these guidelines has made it possible to estimate that a beginner operator reaches the level of proficiency and safety necessary for LAAO once he or she has performed approximately ten cases. Although all of the prostheses available in the Brazilian market follow different requirements and implantation techniques, there is a “group effect” in learning the general technique, which allows for partial sharing of the learning curves between prosteses.^39,41^

##### 6.2.2.12. Transcatheter Aortic Valve Replacement

AoS currently shows a growing prevalence due to increased life expectancy and consequent population aging. Currently, the most common cause of AoS is aortic calcification, which mainly affects elderly patients, being observed at a prevalence of 4.6% in individuals over 75 years old.^42-44^ TAVR has become an option for surgical valve replacement in select cases following careful assessment of life expectancy, degree of frailty, and aortic valve anatomy.^43,45-52^

Interventional cardiologists who perform this procedure should have extensive knowledge of the results, limitations, and complications of medical therapy, aortic valve replacement, stimulation with a pacemaker, and TAVR itself.^45-52^ It is recommended that TAVR involve a multidisciplinary program that includes the contributions of a clinical cardiologist, an echocardiographist, a radiologist, and a cardiac surgeon. The following are, furthermore, recommended for the operator in accordance with the joint resolution established between the SBHCI and the Brazilian Society of Cardiovascular Surgery (Sociedade Brasileira de Cirurgia Cardiovascular - SBCCV) in January 2017:

- A certificate in the area of hemodynamics and interventional cardiology.
- Participation in theoretical didactic sessions, with a minimum course load of 24 hours, in courses administrated or recognized by the SBHCI and the SBCCV.
- Participation in training sessions with simulators, with a minimum course load of 2 hours.
- Participation, as an observer, in at least two TAVR procedures in training centers accredited by Brazilian medical societies or care centers that regularly contribute to the Brazilian Registry of Transcatheter Aortic Valve Bioprosthesis Implantation, as certified by the coordinator of the Center.
- Participation in discussions of clinical cases related to TAVR procedures, with a minimum course load of four hours, in training centers accredited by the medical societies, as certified by the coordinator of the Center.
- In transfemoral procedures, perform a minimum of five procedures over the past two years, as a first operator, under the supervision of a qualified specialist (proctor).
- Proficiency and autonomy attested by a supervisor specialist accredited by the SBCCV and the SBHCI (at the supervisor’s discretion, training may be extended to a higher number of supervised cases).
- Contribution to the Brazilian Registry of Catheter Valve Therapy during at least the first 25 procedures, performed without supervision.

Candidates for TAVR Qualification Certificates must submit the proof of training documents to the Certification Committee of the SBHCI and the SBCCV, in order to verify that they have met the previously described requirements.

Candidates who undergo training in TAVR abroad may be certified, provided that they have met the requirements established here and that they present documentation which proves that they have completed training, with the signature of the technical manager of the institution.

###### 6.2.2.12.1. Basic Knowledge

- Natural history and etiology of aortic valve stenosis.
- Hemodynamics of left ventricular outlet obstructions.
- Interpretation and familiarity with different imaging exams: TTE, TEE, CT, MR, and angiography.
- Knowledge of current guidelines for aortic valve stenosis.
- Therapeutic options.
- Indications for intervention.

###### 6.2.2.12.2. Interventional Cardiologists’ Abilities

- Hemodynamic interpretation of pressure curves.
- Choice of vascular access.
- Introducers, wires, and catheters utilized.
- Angiographic projections for performing the procedure.
- Pre-procedure assessment of CT and other exams for procedure planning.
- Right ventricular pacemaker during intervention (rapid pacing).
- Crossing of the aortic valve orifice with diverse guidewires for positioning inside the left ventricle.
- Performance of balloon aortic valvuloplasty.
- Recognition and rapid management of complications (vascular complications, coronary occlusion, stroke, cardiac tamponade, hemodynamic collapse, iatrogenic IVC, cardiac perforations, arrhythmias/atrioventricular blocks, etc.).
- Immediate and long-term post-procedural care.

##### 6.2.2.13. Transcatheter Mitral Valve Repair

Mitral valve insufficiency, one of the most common acquired valve diseases, frequently affects elderly patients, who present many comorbidities and, at times, are not able to be treated conventionally by mitral valve surgery due to the high operative risk of death and complications.^42,43^ TMVR is a viable option for treating moderate or severe mitral insufficiency, in symptomatic patients, patients with high surgical risks, or inoperable patients, with degenerative or functional etiology, as an alternative to conventional surgical treatment or isolated clinical treatment.^53-59^

Interventional cardiologists who perform this procedure must have specific training in cardiovascular catheterization, required during professional qualification, as well as experience in diagnostic procedures for valve diseases, which are essential to their safety and success.^53-59^ Mastery of the transseptal puncture technique is also necessary, as is knowledge of its anatomical relation with the pulmonary artery, the coronary sinus, the aorta, and other cardiac structures, because, in some cases, accidents may occur during puncture of the interatrial septum. Specific knowledge is also necessary regarding the general characteristics of implantable medical devices utilized by this technique and their appropriate indications, as well as understanding of anticoagulation control and appropriate management of possible complications, such as cardiac tamponade, cardiac or vascular perforation, clip embolization, thrombus formation, infectious endocarditis, cardiac arrhythmias, and other complications.

It is recommended that TMVR involve a multidisciplinary program that includes the contributions of a clinical cardiologist, an echocardiographist, a radiologist, and a cardiac surgeon. Qualification of physicians for mitral clip therapy requires thorough knowledge of normal cardiac anatomy, anatomy of right and left chambers, and, above all, an understanding of anatomical anomalies, their functional repercussions, and the corresponding relative values of therapeutic options. The duration of the learning curve varies significantly in accordance with the operator’s degree of familiarity with procedures for congenital and/or structural heart diseases, as well as the frequency with which the procedure is performed. Although there is no consensus in the literature with respect to the minimum number of cases required in order to complete this learning curve, within the Brazilian context, considering the complexity of the intervention, it is possible to estimate that a beginner operator will reach the level of proficiency and safety necessary for TMVR once he or she has performed approximately ten cases.

###### 6.2.2.13.1. Basic Knowledge

- Natural history and etiology of mitral valve insufficiency.
- Hemodynamics of mitral insufficiency.
- Interpretation and familiarity with different imaging exams: TTE, TEE, CT, MR, and angiography.
- Knowledge of current Brazilian guidelines for treating mitral valve insufficiency.
- Therapeutic options.
- Indications for intervention.

###### 6.2.2.13.2 Interventional Cardiologists’ Abilities

- Hemodynamic interpretation of pressure curves.
- Access management.
- Ability with the introducers, wires, and catheters utilized.
- Angiographic projections for performing the procedure.
- Pre-procedure assessment of TEE for patient selection.
- Recognition and rapid management of complications (vascular complications, stroke, cardiac tamponade, hemodynamic collapse, cardiac perforations, arrhythmias/atrioventricular blocks, etc.).
- Immediate and long-term post-procedural care.

##### 6.2.2.14 Transcatheter Pulmonary Valve Bioprosthesis Implantation

Right ventricular outflow tract dysfunctions are frequently involved in the late postoperative period of right ventricle to pulmonary artery (RV-PA) connection surgeries in patients with tetralogy of Fallot, pulmonary atresia, truncus arteriosus, or any other congenital heart disease in which pulmonary flow must be anatomically restored. In this context, pulmonary insufficiency and, mainly, its association with pulmonary stenosis (double pulmonary valve lesion) may result in dilatation and progressive right ventricular dysfunction, exercise intolerance, potentially severe arrhythmias, and sudden death. Reestablishing pulmonary valve function at an appropriate moment may reverse this process, thus restoring ventricular function and improving symptoms.^60,61^

Surgical replacement of the pulmonary valve requires extracorporeal circulation, which may further aggravate right ventricular function, when it is already compromised.^61^ There are several options and surgical techniques for treating pulmonary insufficiency, including the use of cadaver homografts, valved synthetic conduits, bovine jugular vein grafts, or a bioprosthetic valve implanted directly in the right ventricular outflow tract. With the passing of time, however, all of these conduits or surgically implanted valves present varying degrees of dysfunction characterized by stenosis, accompanied or unaccompanied by insufficiency. It is estimated that after four to five years, 25% of patients who have undergone a homograft implantation will require some type of intervention to increase the longevity of these conduits.^62^ The probability of not needing a conduit replacement is approximately 50% over ten years, with even less favorable figures in small children.^62^

In 2011, the American Heart Association (AHA) published a scientific statement on interventions in congenital heart diseases, in which the recommendation for TPVI was classified as IIa (level of evidence B), stating that, “It is reasonable to consider percutaneous pulmonary valve replacement in a patient with an RV-to-pulmonary artery conduit with associated moderate to severe pulmonary regurgitation or stenosis provided the patient meets inclusion/exclusion criteria for the available valve.”^63^

The TPVI procedure should be performed in a conventional catheterization laboratory or in a hybrid operating room, and the institution should have a group of professionals qualified to treat congenital and structural heart diseases (heart team), made up of a clinical cardiologist, a cardiologist with a certificate in the area of hemodynamics and interventional cardiology, a cardiovascular surgeon, and other professionals directly or indirectly related to the procedure. It is highly recommendable that the operators possess vast experience in diagnostic and therapeutic percutaneous procedures for congenital heart diseases, especially stent implantations in pulmonary arteries. Also necessary are mastery of the coronary catheterization technique and knowledge of the anatomical relation of the origins of coronary arteries with the pulmonary artery, given that, in some cases, release of the pulmonary bioprosthesis might lead to coronary occlusion, which would contraindicate the therapy.

In addition to these prerequisites, it is necessary to possess specific knowledge regarding the general characteristics of catheter-implanted valve prostheses, their appropriate indications in accordance with the underlying congenital heart disease, anticoagulation control, and adequate management of possible complications, such as coronary compression, cardiac or vascular perforation, partial or total rupture of the treated conduit, prosthesis embolization, prosthetic thrombus formation, infectious endocarditis, cardiac arrhythmias, etc.

The specialist physician should possess knowledge of the different surgical strategies used for right ventricular outflow reconstruction, which are necessary to treat complex congenital heart diseases, such as tetralogy of Fallot, pulmonary atresia with IVC, double right ventricular outflow with infundibular pulmonary stenosis, transposition of great arteries with IVC and infundibular pulmonary stenosis, corrected transposition of great arteries with pulmonary stenosis, and common truncus arteriosus.

Concerning, moreover, qualification of physicians, specialists responsible for performing TPVI are required to bear a certificate in the area of hemodynamics and interventional cardiology, duly registered with the CRM in the jurisdiction in which they are professionally active, in accordance with current legislation, and they must have participated in at least ten procedures under the supervision of a qualified physician (proctor), in order to have adequate proficiency and safety for the implantation.

###### 6.2.2.14.1 Basic Knowledge

- Natural history of different types of conduits or valves (synthetic, biological, homografts, valved, or non-valved) utilized for the connection between the right ventricle and the pulmonary trunk (RV-PT).
- Knowledge of the anatomy and physiology of different intra- and extracardiac surgical conduits and tunnels.
- Comprehension of the anatomy and hemodynamics of different surgical procedures used for treating complex congenital heart diseases.
- Knowledge of the effects of pressure and volume overloads on the pulmonary ventricle in patients with complex congenital heart diseases and conduits.
- Indications for intervention.

###### 6.2.2.14.2 Interventional Cardiologists’ Abilities

- Hemodynamic interpretation of complex congenital heart diseases in the hemodynamics laboratory.
- Choice of access.
- Knowledge and abilities with imaging methods for guiding the procedure: echocardiography, CT, MR, and angiography.
- Knowledge and appropriate choice of different catheters, guides, balloons, sheaths, stents, covered stents, and devices available for this procedure and their techniques.
- Assessment of coronary circulation during balloon compression test in the conduit.
- Knowledge of balloon catheters for dilatation.
- Techniques for stent implantations.
- Adequate and safe implantation of pulmonary valve replacement devices.
- Recognition and rapid management of complications (vascular complications, coronary occlusion, stroke, cardiac tamponade, hemodynamic collapse, iatrogenic IVC, cardiac perforations, arrhythmias/atrioventricular blocks, etc.).
- Immediate and long-term post-procedural care.

### 6.3. Extracardiac Interventions

Since their appearance, diagnostic and therapeutic endovascular procedures in the extracardiac vascular bed have historically been performed by interventional cardiologists.

Interventional cardiologists notably possess extensive scientific knowledge about systemic atherosclerotic diseases and the use of anticoagulants and antiplatelet agents, and they also have mastery of the technical procedures for performing angioplasty with stent implantation, using embolic protection filters, and others.^2,3,8,64^

With the expansion and rapid advancement of techniques for peripheral intervention over the past decade, it has become necessary for the SBHCI to accompany the formation and maintenance of training and expertise of its affiliated professionals closely.

#### 6.3.1. Norms for Establishing Hemodynamics and interventional Cardiology Training Centers that Include Extracardiac Interventions

The basic rules are the same as those previously described in this Position Paper. Centers must, additionally, routinely perform extracardiac vascular procedures, in a manner that trainees are able to acquire the theoretical and practical knowledge required for good medical practice. In the literature, there is not a defined number of procedures that establish a service’s competence in this area.

##### 6.3.1.1. Medical Teams

Medical teams must be made up of preceptors and a coordinator, with the same prerequisites previously defined. There should, additionally, be professionals with experience and recognized expertise in extracardiac interventions. The number of vascular procedures necessary for maintaining competence has not been established, and it is controversial in the literature. One motive which justifies this difficulty is that we are dealing with procedures which are less prevalent in all services. It is however, recommended, as an analogy of the proposed volume for maintaining trainee competence, that preceptors perform at least 50 therapeutic endovascular interventions annually.

##### 6.3.1.2. Trainees

The prerequisites and basic obligations of trainees have been previously described. For specific acquisition of competence in extracardiac interventions, trainees must act as first operators in:

- 100 diagnostic exams (including aortographies; angiographies of carotid, vertebral, subclavian, upper-member, lower-member, abdominal, and renal arteries; venous angiographies; cavographies, and pulmonary artery catheterizations).
- 50 endovascular interventions in different peripheral beds (miscellany).
- 12 carotid angioplasties.

##### 6.3.1.3. The Theoretical-Practical Program

- The minimum specific training period is 12 consecutive months, with 30 days of vacation per year, scientific improvement, and participation in congresses and reunions related to the specialization.
- The year of extracardiac training may simultaneously correspond to the second year of specialization at the training center, in the event that the center routinely performs this type of intervention.
- In the event that the training does not routinely perform the extracardiac vascular procedures necessary for the acquisition of abilities on the part of the trainee, specific training may take place by means of improvement courses, sanctioned by the SBHCI, concomitant to or after the second year of specialization.
- The minimum syllabus for trainees in hemodynamics and interventional cardiology has already been described. In relation to specific aspects of training for the peripheral bed, the following are also necessary:
- Mastery of alternative vascular accesses, such as anterograde puncture of the common femoral artery, brachial puncture, radial puncture, fistula puncture, superficial femoral puncture, popliteal puncture, distal vessel puncture in the lower limbs, whether ultrasound-guided or not.
- Comprehensive knowledge of peripheral vascular anatomy.
- Accompanying theoretical discussions of extracardiac cases for one year with preceptors in the specialization or with SBHCI endovascular course coordinators.
- For training in the improvement course, the minimum theoretical course load is 200 hours/year.

#### 6.3.2. Individualized Knowledge for Each Type of Intervention

##### 6.3.2.1. Interventions in Lower Limbs

###### 6.3.2.1.1. Basic Knowledge

- Knowledge of the natural history of atherosclerosis and its manifestations in lower members, differentiating the needs of claudicating patients from those who have critical ischemia, whether acute or chronic.
- Clinical treatment of ischemia of the lower limbs.
- Differentiation of conduct for inflow and outflow diseases.
- Diagnostic exams required for image interpretation.
- Indications for invasive intervention.
- How to proceed with follow-up.

###### 6.3.2.1.2 Interventional Cardiologists’ Abilities

- Definition and execution of the best vascular access: anterograde or retrograde.
- Selection of introducers, sheaths, guides, and catheters.
- Selection of appropriate balloons; whether or not prostheses are necessary and their characteristics, balloon- or self-expandable, coated or uncoated.
- Mastery of management of complications by means of rescue of distal occlusions or embolisms with or without the use of local thrombolysis or perforations with embolotherapy techniques.

##### 6.3.2.2. Aortic Interventions (Aneurysms)

###### 6.3.2.2.1. Basic Knowledge

- Natural history of the disease.
- Differentiation of conduct based on the affected segment of the aorta.
- Diagnostic exams required for image interpretation.
- Indications for invasive intervention and technical knowledge for prevention and treatment of leaks.
- Planning, indication, and management by a multidisciplinary team (heart team).
- How to conduct follow-up.

###### 6.3.2.2.2 Interventional Cardiologists’ Abilities

- Definition of the best vascular access and mastery of totally percutaneous techniques and hemostatic devices.
- Selection of introducers, sheaths, guides, and catheters.
- Selection of endoprostheses and their characteristics.
- Mastery of management of complications by means of rescue of occlusions or perforations with techniques for covered prostheses.

##### 6.3.2.3. Interventions in Renal Arteries

###### 6.3.2.3.1. Basic Knowledge

- Natural history of the disease.
- Differentiation of conduct based on the clinic and anatomy.
- Diagnostic exams required for image interpretation.
- Indications for invasive intervention.
- How to conduct follow-up.

###### 6.3.2.3.2. Interventional Cardiologists’ Abilities

- Definition of best vascular access.
- Selection of introducers, sheaths, guides, and catheters.
- Selection of balloons and stents.
- Mastery of management of complications by means of rescue of occlusions or perforations with embolotherapy techniques.

##### 6.3.2.4. Interventions in Carotid Arteries and Vessels of the Base

###### 6.3.2.4.1. Basic Knowledge

- Natural history of the disease.
- Differentiation of conduct based on the clinic and extra- and intracranial vascular anatomy.
- Diagnostic exams required for image interpretation.
- Indications for invasive intervention.
- How to conduct follow-up.

###### 6.3.2.4.2. Interventional Cardiologists’ Abilities

- Definition of best vascular access.
- Selection of introducers, sheaths, guides, and catheters.
- Selective, atraumatic catheterization of the vessels of the base.
- Selection of the form of cerebral protection, filters, or proximal protection, balloons, and stents.
- Mastery of management of local complications by means of rescue of intracranial occlusions by local thrombolysis and retriever devices.

##### 6.3.2.5. Embolotherapy

###### 6.3.2.5.1. Basic Knowledge

- Identification of which conditions of vascular complications require endovascular intervention for occlusion of the target vessel.
- Diagnostic exams required for image interpretation.
- Indications for invasive intervention.
- How to conduct follow-up.

###### 6.3.2.5.2. Interventional Cardiologists’ Abilities

- Definition of best vascular access.
- Selection of introducers, sheaths, guides, and catheters.
- Selective, atraumatic catheterization of target vessels.
- Correct selection of embolization agent for every necessity.
- Mastery of the use of microcatheters, microguides, coils, particles, plugs, Onyx, and biological glue.

##### 6.3.2.6. Venous Diseases

###### 6.3.2.6.1. Basic Knowledge

- Venous diseases treatable by endovascular treatment, such as thromboses, central occlusions, stenoses, May-Thurner syndrome, and nutcracker syndrome, among others.
- Diagnostic exams required for image interpretation.
- Indications for invasive intervention.
- How to conduct follow-up.

###### 6.3.2.6.2. Interventional Cardiologists’ Abilities

- Selection of introducers, sheaths, guides, and catheters based on the condition to be handled.
- Selection of balloons and stents.
- Knowledge of techniques for chemical and mechanical thrombolysis; indications and management of vena cava filters.

Regarding procedures whose indications and degrees of evidence have fluctuated over the past years, such as pulmonary branch angioplasty and renal denervation, it is necessary for trainees and interventional cardiologists who are already qualified to stay continuously updated.

Finally, interventional cardiologists are encouraged to have knowledge and training for the use of mechanical thrombectomy in the case of an acute ischemic stroke, which may result from both percutaneous procedures performed and separate events, whose limited therapeutic window makes transfer to another center difficult.

## 7. Final Considerations

The great advance recently observed in interventional cardiology is limited not only to percutaneous treatment of the coronary artery disease, but also to the treatment of congenital heart diseases, the extracardiac vascular bed and, above all, structural heart diseases. Establishment and maintenance of training centers are fundamental in order to guarantee that new interventionists acquire the abilities necessary to carry out interventional treatment of diseases that are included in this vast and complex area of cardiology practice, with excellence. In this manner, the SBHCI must assume the coordination of actions and norms that provide for the certification of training centers and new interventionists. The SBHCI must also act as a facilitator of continued medical education in the area of interventional cardiology, with the objective of providing society with professionals whose abilities and responsibilities adequately meet the population’s expectations. This entire process should be periodically revised, and any eventual adaptations should be published in the form of guidelines or recommendations.

## Annex 1

**RADIATION PROTECTION**

Technological advances over the past decades have made it possible for interventional cardiology to expand visibly, promoting diagnosis and therapy of numerous diseases in a less invasive manner and with minimal risks for patients. This area of practice has gone from having a diagnostic perspective to intensely acting to treat cardiovascular conditions, encompassing complex coronary interventions, extracardiac vascular diseases, and congenital and structural heart diseases. In addition, the type and complexity of interventions, as well as the clinical severity of patients have significantly increased.^65^ What is thus observed is an increasing radiation dose used for interventional procedures over the past years.

**Biological Risk of Exposure to Ionizing Radiation in Interventional Cardiology**

Exposure to ionizing radiation in a routine and continuous manner may lead to harmful biological effects on the human body, by direct or indirect action on the cells, causing physiological and/or functional effects on the organs. Studies have shown that exposed professionals have increased risks of cataracts,^66,67^ brain tumors, skin lesions, and hereditary genetic alterations.^68^ Radiation protection measures, both for individuals and for institutional requirements, are thus essential for everyone who works with these agents.

**Institutional Requirements**

It is necessary for the institution where the hemodynamics and interventional cardiology laboratory functions to have the following:

- A medical technician responsible for ensuring all service licensing norms, in compliance with federal and state health legislation.
- A physician specialist in radiodiagnostics, as required by current legislation.
- Personal protective equipment (PPE) in sufficient quantity for the whole team, such as a 0.5 mm lead-equivalent apron, lead glasses with side shields, and thyroid shields.
- Barrier measures, such as lower (“skirt”) and upper (“shield”) screens are also requisites for protection.
- Upkeep of PPE and its respective integrity tests, also on an annual basis (carried out and registered).
- Individual dosimeter.

**Technical Responsibility**

The managing technician has the following responsibilities:

- Be duly qualified and capacitated to exercise this function and ensure the service’s proper functioning.
- Establish a radiation protection program that includes service routines, technique standardization, and specific radiation protection measures.
- Ensure that annual training and qualification of medical, care, and technical teams take place, including radiation protection measures, correct use of PPE, correct use of individual dosimeter, and correct equipment use.
- Nominate subordinate people to assist in the construction and execution of activities that involve the radiation protection program, such as other physicians, physician specialists in radiodiagnostics, radiology technicians, and work safety technicians, i.e., encourage a culture of protection.
- Guarantee preventative maintenance and dosimetry of equipment and a specific quality program for cinefluoroscopic equipment, including performance tests that evaluate precepts of image quality and radiological safety, with the frequency recommended by current legislation.

**Individual Protection Measures**

Individual protection measures include the following:

- All individuals who work with radiation must use the aforementioned PPE (apron, lead glasses with side shield, and thyroid shield).
- The operators, in addition to PPE, must use protective barriers (upper and lower screens) during all procedures.
- No employee should exceed individual dose limits stipulated by current legislation:^69^
  - The average annual effective dose must not exceed 20 mSv during any period of five consecutive years, and it must not exceed 50 mSv during any year.
  - The equivalent annual dose must not exceed 500 mSv for any extremity and 150 mSv for the lens of the eye.

**Quality Control**

Quality control measures include:

- The service should monitor all procedure doses and maintain the records of patient doses for consultation.
- The radiation protection program should establish dose limits for investigation, in the event of patient overexposure, and patients who exceed the program’s established dose must be followed up, with the entire investigation duly registered and attached to his or her medical record. Medical and care teams must also have their doses monitored and registered monthly with the use of a dosimeter, and cases where monthly and/or annual doses are exceeded must also be investigated.
- It is recommended that procedure doses be described in their procedure reports.
- It is recommended that the service implement monthly indicators that refer to radiation exposure and that these be widely disseminated among the members of medical and care teams.

## Annex 2

**FREE AND INFORMED CONSENT AND AUTHORIZATION FORM FOR PERFORMANCE OF THE EXAM/PROCEDURE OF CORONARY CINEANGIOGRAPHY WITH LEFT VENTRICULOGRAPHY, FFR, OR IFR AND ASSESSMENT OF MYOCARDIAL BRIDGING**

By the present Consent and Authorization Form, I, ........................................................................................................, nationality ................................................, a legal adult and able, marital status .........................................................., profession ....................................................., identity document ......................................................., issued by ......................................., CPF ...................................................., resident of ..........................................................................................................................................., city .............................................................., state ....................., date of birth ........../........../.........., parents’ names ........................................................................................................................................., hereby declare that I have received from the Hemodynamics Service, here represented by the physician fully identified below, explanations and warnings concerning the exam/procedure solicited by my clinical physician and that the discussion regarding the nature and extent of the actions necessary for its execution is registered below.

I further declare that it has been explained to me that the exam/procedure cineangiography with left ventriculography, intracoronary ultrasound (ICUS), fractional flow reserve (FFR), or instantaneous free-wave ratio (iFR) and assessment of myocardial bridging will be performed in the hemodynamics laboratory of this hospital institution (corporate name .............................................................................................., CNPJ/MF no. ...................................headquarters....................................................................) and that it consists of punctures in the patient’s skin in order to introduce special catheters, using iodized contrast, with the administration of local anesthesia, sedation, or general anesthesia, at the attending physician’s discretion.

The following text has been read and explained to me in more accessible language by the signing physician: the aim of this exam/procedure is to facilitate diagnosis of obstructions or blocks in arteries that irrigate or supply the cardiac muscle, known as coronary arteries. The exam/procedure consists of the insertion of a fine catheter through an arterial puncture, generally in the inguinal region or the radial artery. Exceptionally, it may be done by dissection/puncture of the brachial artery. Using this fine catheter, iodized contrast, and X-rays, it will be possible to understand the anatomy of the coronary arteries and, consequently, the extent of blocks which limit the free flow of blood to supply the heart, if they are present. In the event of doubts regarding the significance of the coronary artery blocks found, ICUS may be performed through a microcatheter specifically dedicated to this purpose, which will be introduced into the obstructed coronary artery. Diagnostic clarification regarding the severity of the coronary obstruction or block may be further carried out by means of the introduction of a very fine guidewire inside the coronary artery; this is known as FFR or iFR.

I declare that, in the manner explained to me by the physician, I am perfectly able to understand what the exam/procedure which I am to undergo will comprise.

I declare that I am aware that the intended purpose of performing the forenamed exam/procedure may not be achieved, even though the physician and his/her team adopt the best techniques and employ all of the scientific means and resources available.

I am also aware that the exam/procedure involves risks, and I have received all the pertinent information regarding possible complications due to known and unknown causes, including death, stroke, myocardial infarction, cardiac arrhythmia, acute pulmonary edema, anaphylactic shock, varying types and degrees of infection, allergies and/or reactions to contrast, bleedings, hematomas, renal insufficiency, vascular and hemodynamic complications, perforation of cardiac chambers or vessels, and loss of limbs and/or their function, in addition to the risks inherent in anesthesia and the use of diverse instruments and equipment itself. It has also been explained to me that these adverse reactions and infrequent, occurring in less than 2% of cases, but they may be aggravated when associated with other patient personal factors, which include underlying diseases, previous heart surgery, prior history of allergies, uncontrolled arterial hypertension, tobacco use, alcoholism, diabetes, obesity, renal insufficiency, cerebrovascular disease, prolonged hospitalization, liver failure, heart disease, atherosclerotic disease, cancer, severe malnutrition, and advanced age, which are the most common.

It has also been explained to me that the catheters and prostheses used are subjected to previous tests, but that they may present defects or even fracture, causing adverse reactions and varying types and degrees of injury, including the possibility of requiring surgery to remove them.

It has been reiterated to me that no guarantees or assurances are given with respect to the results expected from the proposed exam/procedure. I am aware that, in executing the proposed exam/procedure, the hemodynamicist and his/her team will be present, and it will be possible to solicit the presence of other specialists, as well as observers from the manufacturer of the equipment and material used. My signature at the end of this consent form authorizes the participation of these professionals and grants them full access to records and medical information related to my case. I have also been informed that, should it be necessary, bed restraint may be performed, as prescribed by the physician, and that I will be under the surveillance of the nursing staff.

I further declare that I have had the opportunity to read this document carefully and also to listen attentively to the physician’s reading of all the information presented herein, and I was able to clarify all of my doubts regarding the risks involved and the possible consequences of not performing the suggested treatment, as well as the possible existence of alternative procedures, and my doubts have been satisfactorily addressed and answered. I am aware that there is a possibility of alteration of the original procedure over the course of the aforementioned exam/procedure and/or new procedure or surgery for the sake of continuity of treatment in a different region from the one originally designated, which, if applicable, will be evaluated, decided upon, and performed, by the medical professional in charge, with which I now agree and hereby authorize.

All my questions having been answered and having understood the procedure in and of itself, as well as its real repercussions, I sign this CONSENT FORM, in the presence of Dr. ................................................................................................................, who, in this act, represents the Hemodynamic Medical Service, together with 2 (two) witnesses, expressly and voluntarily declaring my authorization for the exam/procedure of coronary cineangiography with left ventriculography, FFR, or iFR and assessment of myocardial bridging, consenting, furthermore, to the performance of the proposed exam/procedure as well as its preparatory acts; the physicians are hereby authorized to performed additional procedures and therapeutic interventions that may become necessary due to unforeseen conditions which may occur during the course of the exam/procedure, remaining always at the discretion and judgment of the attending physician.

For the purpose of promoting scientific development, by signing the present Consent Form, I further agree and authorize the performance of photography, video recording, or televised transmission of the proposed exam/procedure, being assured that my identity will not be revealed. I also authorize the examination of any organ or tissue eventually removed, which may be treated by the medical team and/or the hospital for medical, scientific, and educational purposes.

Finally, I consent to the eventual possibility of a blood transfusion, should this procedure be directly linked to the treatment mentioned in this consent and authorization form or resulting from it.

If, during or immediately after the performance of the exam/procedure which I am to undergo, I am not in full possession of my physical and/or mental abilities, I authorize my legal representative ................................................................................................................................, nationality ...................................................., marital status ......................................................., profession ....................................................., identity document .................................................., issued by ................................., CPF ..............................................................., resident of ......................................................................................................................................, city ................................................................, state ................., date of birth ........../........../.........., parents’ names........................................................................................................................................., relationship to patient ............................................................, to make decisions in my name.

In this manner, I offer my full and free consent, and I authorize the performance of the exam/procedure of coronary cineangiography with left ventriculography, FFR, or iFR and assessment of myocardial bridging, explained herein, to be administered by the signing physician and his/her related medical team.

In conclusion, I certify, that I have signed two copies of the present Form, one of which has been given to me.

**Table:**

Center Location: ..........................................	
Date: ........../........../..........	
Patient's Name: ......................................................	Signature: ..................................................
	
Representative: ............................................	Signature: ...................................................
Physician's Name: ...................................................	
Signature: ..................................................	CRM nº.: ..........................
	
Witnesses:	
Name: ........................................................	Name: ........................................................

## Annex 3

**FREE AND INFORMED CONSENT AND AUTHORIZATION FORM FOR PERFORMANCE OF THE PROCEDURE OF CORONARY ANGIOPLASTY WITH OR WITHOUT STENT IMPLANTATIONS**

By the present Consent and Authorization Form, I, ........................................................................................................, nationality ................................................, a legal adult and able, marital status .........................................................., profession ....................................................., identity document ......................................................., issued by ......................................., CPF ...................................................., resident of ..........................................................................................................................................., city .............................................................., state ....................., date of birth ........../........../.........., parents’ names ........................................................................................................................................., hereby declare that I have received from the Hemodynamics Service, here represented by the physician fully identified below, explanations and warnings concerning the procedure solicited by my clinical physician and that the discussion regarding the nature and extent of the actions necessary for its execution is registered below.

I further declare that it has been explained to me that the procedure of coronary angioplasty with or without stent implantations will be performed in the hemodynamics laboratory of this hospital institution (corporate name .............................................................................................., CNPJ/MF no. ...................................headquarters....................................................................) and that it consists of punctures in the patient’s skin in order to introduce special catheters, using iodized contrast, with the administration of local anesthesia, sedation, or general anesthesia, at the attending physician’s discretion.

The following text has been read and explained to me in more accessible language by the signing physician: the objective of this procedure is to treat obstructions or blocks in the arteries that irrigate or supply the heart, known as coronary arteries. The procedure consists of the insertion of a fine catheter through a puncture in the radial artery or the inguinal region, at the physician’s discretion. By means of this fine catheter, iodized contrast, and X-rays, the obstructed or blocked coronary artery(ies) will be approached with one or more very fine guidewires, and a balloon catheter and a very small metallic mesh called a “stent” may be utilized to unblock the artery. Depending on the type of block, other devices may be used to treat the coronary artery(ies), such as the Rotablator, which consists of a very small burr used to unblock extremely calcified (hard) arteries, and a balloon catheter that cuts the plaque or the coronary artery block may also be used. In addition to treatment with the diverse materials mentioned, medications that help decrease the occurrence of clots, such as glycoprotein IIb/IIIa antagonists or other antiplatelet agents, may be used. Moderate or angiographically indeterminate coronary artery blocks may be assessed by means of a very fine guidewire dedicated to assessing these types of blocks. To facilitate the success of this treatment, intravascular ultrasound study, by means of a microcatheter dedicated to this purpose, may be introduced into the coronary artery to guide stent placement. In the event of a medical emergency, a balloon catheter with a diameter similar to that of the aorta may be used and positioned in the descending thoracic portion to facilitate the filling of coronary arteries.

I declare that, in the manner explained to me by the physician, I am perfectly able to understand what the procedure which I am to undergo will comprise.

I declare that I am aware that the intended purpose of performing the forenamed procedure may not be achieved, even though the physician and his/her team adopt the best techniques and employ all of the scientific means and resources available.

I am also aware that the procedure involves risks, and I have received all the pertinent information regarding possible complications due to known and unknown causes, including death, stroke, myocardial infarction, cardiac arrhythmia, acute pulmonary edema, anaphylactic shock, varying types and degrees of infection, allergies and/or reactions to contrast, bleedings, hematomas, renal insufficiency, vascular and hemodynamic complications, perforation of cardiac chambers or vessels, and loss of limbs and/or their function, in addition to the risks inherent in anesthesia and the use of diverse instruments and equipment itself. It has also been explained to me that these adverse reactions and infrequent, occurring in less than 2% of cases, but they may be aggravated when associated with other patient personal factors, which include underlying diseases, previous heart surgery, prior history of allergies, uncontrolled arterial hypertension, tobacco use, alcoholism, diabetes, obesity, renal insufficiency, cerebrovascular disease, prolonged hospitalization, liver failure, heart disease, atherosclerotic disease, cancer, severe malnutrition, and advanced age, which are the most common.

It has also been explained to me that the catheters and prostheses used are subjected to previous tests, but that they may present defects or even fracture, causing adverse reactions and varying types and degrees of injury, including the possibility of requiring surgery to remove them.

It has been reiterated to me that no guarantees or assurances are given with respect to the results expected from the proposed procedure. I am aware that, in executing the proposed procedure, the hemodynamicist and his/her team will be present, and it will be possible to solicit the presence of other specialists, as well as observers from the manufacturer of the equipment and material used. My signature at the end of this consent form authorizes the participation of these professionals and grants them full access to records and medical information related to my case. I have also been informed that, should it be necessary, bed restraint may be performed, as prescribed by the physician, and that I will be under the surveillance of the nursing staff.

I further declare that I have had the opportunity to read this document carefully and also to listen attentively to the physician’s reading of all the information presented herein, and I was able to clarify all of my doubts regarding the risks involved and the possible consequences of not performing the suggested treatment, as well as the possible existence of alternative procedures, and my doubts have been satisfactorily addressed and answered. I am aware that there is a possibility of alteration of the original procedure over the course of the aforementioned procedure and/or new procedure or surgery for the sake of continuity of treatment in a different region from the one originally designated, which, if applicable, will be evaluated, decided upon, and performed, by the medical professional in charge, with which I now agree and hereby authorize.

All my questions having been answered and having understood the procedure in and of itself, as well as its real repercussions, I sign this CONSENT FORM, in the presence of Dr. ..............................................................................................................., who, in this act, represents the Hemodynamic Medical Service, together with 2 (two) witnesses, expressly and voluntarily declaring my authorization for the procedure of coronary angioplasty with or without stent implantations, consenting, furthermore, to the performance of the proposed procedure as well as its preparatory acts; the physicians are hereby authorized to performed additional procedures and therapeutic interventions that may become necessary due to unforeseen conditions which may occur during the course of the procedure, remaining always at the discretion and judgment of the attending physician. Finally, I consent to the eventual possibility of a blood transfusion, should this procedure be directly linked to the treatment mentioned in this consent and authorization form or resulting from it.

If, during or immediately after the performance of the procedure which I am to undergo, I am not in full possession of my physical and/or mental abilities, I authorize my legal representative ................................................................................................................................, nationality ...................................................., marital status ......................................................., profession ....................................................., identity document .................................................., issued by ................................., CPF ..............................................................., resident of ......................................................................................................................................, city ................................................................, state ................., date of birth ........../........../.........., parents’ names........................................................................................................................................., relationship to patient ............................................................, to make decisions in my name.

In this manner, I offer my full and free consent, and I authorize the performance of the procedure of coronary angioplasty with or without stent implantations, explained herein, to be administered by the signing physician and his/her related medical team.

In conclusion, I certify, that I have signed two copies of the present Form, one of which has been given to me.

**Table:**

Center Location: ..........................................	
Date: ........../........../..........	
Patient's Name: ......................................................	Signature: ..................................................
	
Representative: ............................................	Signature: ...................................................
	
Physician's Name: ...................................................	CRM nº.: ..........................
Signature: ..................................................	
	
Witnesses:	
Name: ........................................................ Name: ........................................................	

## Annex 4

**FREE AND INFORMED CONSENT AND AUTHORIZATION FORM FOR PERFORMANCE OF THE PROCEDURE OF BALLOON MITRAL VALVULOPLASTY**

By the present Consent and Authorization Form, I, ........................................................................................................, nationality ................................................, a legal adult and able, marital status .........................................................., profession ....................................................., identity document ......................................................., issued by ......................................., CPF ...................................................., resident of ..........................................................................................................................................., city .............................................................., state ....................., date of birth ........../........../.........., parents’ names ........................................................................................................................................., hereby declare that I have received from the Hemodynamics Service, here represented by the physician fully identified below, explanations and warnings concerning the procedure solicited by my clinical physician and that the discussion regarding the nature and extent of the actions necessary for its execution is registered below.

I further declare that it has been explained to me that the procedure balloon mitral valvuloplasty will be performed in the hemodynamics laboratory of this hospital institution (corporate name ..........................................................................................................., CNPJ/MF no. ...................................headquarters....................................................................) and that it consists of punctures in the patient’s skin in order to introduce special catheters, using iodized contrast, with the administration of local anesthesia, sedation, or general anesthesia, at the attending physician’s discretion.

The following text has been read and explained to me in more accessible language by the signing physician: the aim of this procedure is to treat narrowing or severe stenosis of the mitral valve by means of a balloon catheter dedicated to this purpose. The procedure consists of puncture in a deep vein and an artery in the inguinal region. Through the femoral vein, from the right atrium, a puncture will be made in the interatrial septum using a special needle. This puncture may be guided by fluoroscopy (X-rays) with or without transesophageal echocardiography. Subsequently, with the aid of a special guidewire, a dedicated balloon catheter will be positioned in the inside of the left atrium. Following predefined maneuvers, the balloon catheter will be positioned in the mitral valve and inflated. Following angiographic control and pressure measurements, simultaneously performed in the left atrium and the left ventricle, the latter by means of a catheter positioned through arterial access, the procedure will be concluded with the removal of the instruments.

I declare that, in the manner explained to me by the physician, I am perfectly able to understand what the procedure which I am to undergo will comprise.

I declare that I am aware that the intended purpose of performing the forenamed procedure may not be achieved, even though the physician and his/her team adopt the best techniques and employ all of the scientific means and resources available.

I am also aware that the procedure involves risks, and I have received all the pertinent information regarding possible complications due to known and unknown causes, including death, stroke, myocardial infarction, cardiac arrhythmia, acute pulmonary edema, anaphylactic shock, varying types and degrees of infection, allergies and/or reactions to contrast, bleedings, hematomas, renal insufficiency, vascular and hemodynamic complications, perforation of cardiac chambers or vessels, and loss of limbs and/or their function, in addition to the risks inherent in anesthesia and the use of diverse instruments and equipment itself. It has also been explained to me that these adverse reactions and infrequent, occurring in less than 2% of cases, but they may be aggravated when associated with other patient personal factors, which include underlying diseases, previous heart surgery, prior history of allergies, uncontrolled arterial hypertension, tobacco use, alcoholism, diabetes, obesity, renal insufficiency, cerebrovascular disease, prolonged hospitalization, liver failure, heart disease, atherosclerotic disease, cancer, severe malnutrition, and advanced age, which are the most common.

It has also been explained to me that the catheters and prostheses used are subjected to previous tests, but that they may present defects or even fracture, causing adverse reactions and varying types and degrees of injury, including the possibility of requiring surgery to remove them.

It has been reiterated to me that no guarantees or assurances are given with respect to the results expected from the proposed procedure. I am aware that, in executing the proposed procedure, the hemodynamicist and his/her team will be present, and it will be possible to solicit the presence of other specialists, as well as observers from the manufacturer of the equipment and material used. My signature at the end of this consent form authorizes the participation of these professionals and grants them full access to records and medical information related to my case. I have also been informed that, should it be necessary, bed restraint may be performed, as prescribed by the physician, and that I will be under the surveillance of the nursing staff.

I further declare that I have had the opportunity to read this document carefully and also to listen attentively to the physician’s reading of all the information presented herein, and I was able to clarify all of my doubts regarding the risks involved and the possible consequences of not performing the suggested treatment, as well as the possible existence of alternative procedures, and my doubts have been satisfactorily addressed and answered. I am aware that there is a possibility of alteration of the original procedure over the course of the aforementioned procedure and/or new procedure or surgery for the sake of continuity of treatment in a different region from the one originally designated, which, if applicable, will be evaluated, decided upon, and performed, by the medical professional in charge, with which I now agree and hereby authorize.

All my questions having been answered and having understood the procedure in and of itself, as well as its real repercussions, I sign this CONSENT FORM, in the presence of Dr. ................................................................................................................, who, in this act, represents the Hemodynamic Medical Service, together with 2 (two) witnesses, expressly and voluntarily declaring my authorization for the procedure of balloon mitral valvuloplasty, consenting, furthermore, to the performance of the proposed procedure as well as its preparatory acts; the physicians are hereby authorized to performed additional procedures and therapeutic interventions that may become necessary due to unforeseen conditions which may occur during the course of the procedure, remaining always at the discretion and judgment of the attending physician. Finally, I consent to the eventual possibility of a blood transfusion, should this procedure be directly linked to the treatment mentioned in this consent and authorization form or resulting from it.

If, during or immediately after the performance of the procedure which I am to undergo, I am not in full possession of my physical and/or mental abilities, I authorize my legal representative ................................................................................................................................, nationality ...................................................., marital status ......................................................., profession ....................................................., identity document .................................................., issued by ................................., CPF ..............................................................., resident of ......................................................................................................................................, city ................................................................, state ................., date of birth ........../........../.........., parents’ names........................................................................................................................................., relationship to patient ............................................................, to make decisions in my name.

In this manner, I offer my full and free consent, and I authorize the performance of the procedure of balloon mitral valvuloplasty, explained herein, to be administered by the signing physician and his/her related medical team.

In conclusion, I certify, that I have signed two copies of the present Form, one of which has been given to me.

**Table:**

Center Location: ..........................................	
Date: ........../........../..........	
Patient's Name: ......................................................	Signature: ..................................................
	
Representative: ............................................	Signature: ...................................................
	
Physician's Name: ...................................................	CRM nº.: ..........................
	
Witnesses:	
	
Name: ........................................................ Name: ........................................................	

## Annex 5

**FREE AND INFORMED CONSENT AND AUTHORIZATION FORM FOR PERFORMANCE OF THE PROCEDURE OF TRANSCATHETER MITRAL VALVE REPAIR**

By the present Consent and Authorization Form, I, ........................................................................................................, nationality ................................................, a legal adult and able, marital status .........................................................., profession ....................................................., identity document ......................................................., issued by ......................................., CPF ...................................................., resident of ..........................................................................................................................................., city .............................................................., state ....................., date of birth ........../........../.........., parents’ names ........................................................................................................................................., hereby declare that I have received from the Hemodynamics Service, here represented by the physician fully identified below, explanations and warnings concerning the procedure solicited by my clinical physician and that the discussion regarding the nature and extent of the actions necessary for its execution is registered below.

I further declare that it has been explained to me that the procedure transcatheter mitral valve repair will be performed in the hemodynamics laboratory of this hospital institution (corporate name ........................................................................................................., CNPJ/MF no. ...................................headquarters....................................................................) and that it consists of punctures in the patient’s skin in order to introduce special catheters, using iodized contrast, with the administration of local anesthesia, sedation, or general anesthesia, at the attending physician’s discretion.

The following text has been read and explained to me in more accessible language by the signing physician: the aim of this procedure is to repair insufficiency or regurgitation of the native mitral valve by means of the placement of a small clip (clamp) in the diseased valve by means of a specific catheter introduced in the inguinal region. This procedure has been recommended by a multidisciplinary team of specialists for patients who have contraindications to cardiac valve repair surgery. Device placement is carried out by means of the insertion of a catheter in the inguinal region to the right atrium of the heart. A puncture will be made in the interatrial septum (the septum that separates the right and left atria), in order to make it possible to position the device containing the clip. The entire procedure is guided by X-ray and mainly by transesophageal echocardiography, which will aid the medical team in the correct positioning and safe release of the mitral valve clip. Once the device has been successfully released, the catheter will be removed and maneuvers will be performed to contain eventual bleedings in the puncture site, and the procedure will be concluded.

I declare that, in the manner explained to me by the physician, I am perfectly able to understand what the procedure which I am to undergo will comprise.

I declare that I am aware that the intended purpose of performing the forenamed procedure may not be achieved, even though the physician and his/her team adopt the best techniques and employ all of the scientific means and resources available.

I am also aware that the procedure involves risks, and I have received all the pertinent information regarding possible complications due to known and unknown causes, including death, stroke, myocardial infarction, cardiac arrhythmia, acute pulmonary edema, anaphylactic shock, varying types and degrees of infection, allergies and/or reactions to contrast, bleedings, hematomas, renal insufficiency, vascular and hemodynamic complications, perforation of cardiac chambers or vessels, and loss of limbs and/or their function, in addition to the risks inherent in anesthesia and the use of diverse instruments and equipment itself. It has also been explained to me that these adverse reactions and infrequent, occurring in less than 2% of cases, but they may be aggravated when associated with other patient personal factors, which include underlying diseases, previous heart surgery, prior history of allergies, uncontrolled arterial hypertension, tobacco use, alcoholism, diabetes, obesity, renal insufficiency, cerebrovascular disease, prolonged hospitalization, liver failure, heart disease, atherosclerotic disease, cancer, severe malnutrition, and advanced age, which are the most common.

It has also been explained to me that the catheters and prostheses used are subjected to previous tests, but that they may present defects or even fracture, causing adverse reactions and varying types and degrees of injury, including the possibility of requiring surgery to remove them.

It has been reiterated to me that no guarantees or assurances are given with respect to the results expected from the proposed procedure. I am aware that, in executing the proposed procedure, the hemodynamicist and his/her team will be present, and it will be possible to solicit the presence of other specialists, as well as observers from the manufacturer of the equipment and material used. My signature at the end of this consent form authorizes the participation of these professionals and grants them full access to records and medical information related to my case. I have also been informed that, should it be necessary, bed restraint may be performed, as prescribed by the physician, and that I will be under the surveillance of the nursing staff.

I further declare that I have had the opportunity to read this document carefully and also to listen attentively to the physician’s reading of all the information presented herein, and I was able to clarify all of my doubts regarding the risks involved and the possible consequences of not performing the suggested treatment, as well as the possible existence of alternative procedures, and my doubts have been satisfactorily addressed and answered. I am aware that there is a possibility of alteration of the original procedure over the course of the aforementioned procedure and/or new procedure or surgery for the sake of continuity of treatment in a different region from the one originally designated, which, if applicable, will be evaluated, decided upon, and performed, by the medical professional in charge, with which I now agree and hereby authorize.

All my questions having been answered and having understood the procedure in and of itself, as well as its real repercussions, I sign this CONSENT FORM, in the presence of Dr. ................................................................................................................, who, in this act, represents the Hemodynamic Medical Service, together with 2 (two) witnesses, expressly and voluntarily declaring my authorization for the procedure of transcatheter mitral valve repair, consenting, furthermore, to the performance of the proposed procedure as well as its preparatory acts; the physicians are hereby authorized to performed additional procedures and therapeutic interventions that may become necessary due to unforeseen conditions which may occur during the course of the procedure, remaining always at the discretion and judgment of the attending physician. Finally, I consent to the eventual possibility of a blood transfusion, should this procedure be directly linked to the treatment mentioned in this consent and authorization form or resulting from it.

If, during or immediately after the performance of the procedure which I am to undergo, I am not in full possession of my physical and/or mental abilities, I authorize my legal representative ................................................................................................................................, nationality ...................................................., marital status ......................................................., profession ....................................................., identity document .................................................., issued by ................................., CPF ..............................................................., resident of ......................................................................................................................................, city ................................................................, state ................., date of birth ........../........../.........., parents’ names........................................................................................................................................., relationship to patient ............................................................, to make decisions in my name.

In this manner, I offer my full and free consent, and I authorize the performance of the procedure of transcatheter mitral valve repair, explained herein, to be administered by the signing physician and his/her related medical team.

In conclusion, I certify, that I have signed two copies of the present Form, one of which has been given to me.

**Table:**

Center Location: ..........................................	
Date: ........../........../..........	
Patient's Name: ......................................................	Signature: ..................................................
	
Representative: .................................	Signature: ...................................................
	
Physician's Name: ...................................................	CRM nº.: ..........................
Signature: ..................................................	
	
Witnesses:	
Name: ........................................................ Name: ........................................................	

## Annex 6

**FREE AND INFORMED CONSENT AND AUTHORIZATION FORM FOR PERFORMANCE OF THE PROCEDURE OF PERCUTANEOUS LEFT ATRIAL APPENDAGE CLOSURE**

By the present Consent and Authorization Form, I, ........................................................................................................, nationality ................................................, a legal adult and able, marital status .........................................................., profession ....................................................., identity document ......................................................., issued by ......................................., CPF ...................................................., resident of ..........................................................................................................................................., city .............................................................., state ....................., date of birth ........../........../.........., parents’ names ........................................................................................................................................., hereby declare that I have received from the Hemodynamics Service, here represented by the physician fully identified below, explanations and warnings concerning the exam/procedure solicited by my clinical physician and that the discussion regarding the nature and extent of the actions necessary for its execution is registered below.

I further declare that it has been explained to me that the exam/procedure percutaneous left atrial appendage closure will be performed in the hemodynamics laboratory of this hospital institution (corporate name .............................................................................................., CNPJ/MF no. ...................................headquarters....................................................................) and that it consists of punctures in the patient’s skin in order to introduce special catheters, using iodized contrast, with the administration of local anesthesia, sedation, or general anesthesia, at the attending physician’s discretion.

The following text has been read and explained to me in more accessible language by the signing physician: the aim of this procedure is to close the left atrial appendage (LAA) by means of implantation or release of a device that will block the LAA off from the rest of the heart in order to decrease the chance of clots forming and provoking a stroke. This is done by means of a puncture in the vein in the inguinal region, through which a catheter will be inserted to the heart. Subsequently, a puncture will be made in the interatrial septum (the septum that separates the right and left atria) in order to allow for positioning of the device in the LAA. The entire procedure is guided by X-ray and mainly by transesophageal echocardiography, which will aid the medical team in the correct positioning and safe release of the device in the LAA. Once the device has been successfully released, the catheter will be removed and the procedure will be concluded.

I declare that, in the manner explained to me by the physician, I am perfectly able to understand what the procedure which I am to undergo will comprise.

I declare that I am aware that the intended purpose of performing the forenamed procedure may not be achieved, even though the physician and his/her team adopt the best techniques and employ all of the scientific means and resources available.

I am also aware that the procedure involves risks, and I have received all the pertinent information regarding possible complications due to known and unknown causes, including death, stroke, myocardial infarction, cardiac arrhythmia, acute pulmonary edema, anaphylactic shock, varying types and degrees of infection, allergies and/or reactions to contrast, bleedings, hematomas, renal insufficiency, vascular and hemodynamic complications, perforation of cardiac chambers or vessels, and loss of limbs and/or their function, in addition to the risks inherent in anesthesia and the use of diverse instruments and equipment itself. It has also been explained to me that these adverse reactions and infrequent, occurring in less than 2% of cases, but they may be aggravated when associated with other patient personal factors, which include underlying diseases, previous heart surgery, prior history of allergies, uncontrolled arterial hypertension, tobacco use, alcoholism, diabetes, obesity, renal insufficiency, cerebrovascular disease, prolonged hospitalization, liver failure, heart disease, atherosclerotic disease, cancer, severe malnutrition, and advanced age, which are the most common.

It has also been explained to me that the catheters and prostheses used are subjected to previous tests, but that they may present defects or even fracture, causing adverse reactions and varying types and degrees of injury, including the possibility of requiring surgery to remove them.

It has been reiterated to me that no guarantees or assurances are given with respect to the results expected from the proposed procedure. I am aware that, in executing the proposed procedure, the hemodynamicist and his/her team will be present, and it will be possible to solicit the presence of other specialists, as well as observers from the manufacturer of the equipment and material used. My signature at the end of this consent form authorizes the participation of these professionals and grants them full access to records and medical information related to my case. I have also been informed that, should it be necessary, bed restraint may be performed, as prescribed by the physician, and that I will be under the surveillance of the nursing staff.

I further declare that I have had the opportunity to read this document carefully and also to listen attentively to the physician’s reading of all the information presented herein, and I was able to clarify all of my doubts regarding the risks involved and the possible consequences of not performing the suggested treatment, as well as the possible existence of alternative procedures, and my doubts have been satisfactorily addressed and answered. I am aware that there is a possibility of alteration of the original procedure over the course of the aforementioned exam/procedure and/or new procedure or surgery for the sake of continuity of treatment in a different region from the one originally designated, which, if applicable, will be evaluated, decided upon, and performed, by the medical professional in charge, with which I now agree and hereby authorize.

All my questions having been answered and having understood the procedure in and of itself, as well as its real repercussions, I sign this CONSENT FORM, in the presence of Dr. ................................................................................................................, who, in this act, represents the Hemodynamic Medical Service, together with 2 (two) witnesses, expressly and voluntarily declaring my authorization for the exam/procedure of percutaneous left atrial appendage closure, consenting, furthermore, to the performance of the proposed procedure as well as its preparatory acts; the physicians are hereby authorized to performed additional procedures and therapeutic interventions that may become necessary due to unforeseen conditions which may occur during the course of the procedure, remaining always at the discretion and judgment of the attending physician. Finally, I consent to the eventual possibility of a blood transfusion, should this procedure be directly linked to the treatment mentioned in this consent and authorization form or resulting from it.

If, during or immediately after the performance of the procedure which I am to undergo, I am not in full possession of my physical and/or mental abilities, I authorize my legal representative ................................................................................................................................, nationality ...................................................., marital status ......................................................., profession ....................................................., identity document .................................................., issued by ................................., CPF ..............................................................., resident of ......................................................................................................................................, city ................................................................, state ................., date of birth ........../........../.........., parents’ names........................................................................................................................................., relationship to patient ............................................................, to make decisions in my name.

In this manner, I offer my full and free consent, and I authorize the performance of the procedure of percutaneous left atrial appendage closure, explained herein, to be administered by the signing physician and his/her related medical team.

In conclusion, I certify, that I have signed two copies of the present Form, one of which has been given to me.

**Table:**

Center Location: ..........................................	
Date: ........../........../..........	
Patient's Name: ......................................................	Signature: ..................................................
	
Representative: ............................................	Signature: ...................................................
	
Physician's Name: ...................................................	CRM nº.: ..........................
Signature: ..................................................	
	
Witnesses:	
Name: ........................................................ Name: ........................................................	

## Annex 7

**FREE AND INFORMED CONSENT AND AUTHORIZATION FORM FOR PERFORMANCE OF THE PROCEDURE OF TRANSCATHETER AORTIC VALVE REPLACEMENT (TAVR)**

By the present Consent and Authorization Form, I, ........................................................................................................, nationality ................................................, a legal adult and able, marital status .........................................................., profession ....................................................., identity document ......................................................., issued by ......................................., CPF ...................................................., resident of ..........................................................................................................................................., city .............................................................., state ....................., date of birth ........../........../.........., parents’ names ........................................................................................................................................., hereby declare that I have received from the Hemodynamics Service, here represented by the physician(s), who are completely identified below, as well as the team known as the “Heart Team,” composed of a clinical cardiology, cardiovascular surgeon, and interventional cardiologist, all the information, explanations, and warnings concerning the exam/procedure solicited by my clinical physician and that the discussion regarding the nature and extent of the actions necessary for its execution is registered below.

I further declare that it has been explained to me that the procedure transcatheter aortic valve implantation (TAVI)  will be performed in the hemodynamics laboratory of this hospital institution (corporate name .............................................................................................., CNPJ/MF no. ...................................headquarters....................................................................) and that it consists of punctures in the patient’s skin in order to introduce special catheters, using iodized contrast, with the administration of local anesthesia, sedation, or general anesthesia, at the attending physician’s discretion.

The following text has been read and explained to me in more accessible language by one of the signing physicians: the aim of this procedure is to treat narrowing or severe stenosis of the aortic valve by means of transcatheter implantation of a biological prosthesis. The valve is positioned using a delivery system. This delivery system helps position the process through the narrow aortic valve. With the heart beating, the prosthesis is released in the site, pushing the leaflets of the diseased valve against the aorta. The new prosthesis will, thus, function in the place of the valve that was diseased, without requiring the latter’s removal. The delivery system is removed and some tests are performed before closing the small incision in the inguinal region. In addition to angiography, the procedure is guided by echocardiography. Depending on each case, dilation may be performed with a special balloon catheter before or after release of the prosthesis. A temporary pacemaker lead will be placed in the right ventricle and it may remain in the patient after the procedure if indicated by the physician.

I declare that, in the manner explained to me by the physician, I am perfectly able to understand what the procedure which I am to undergo will comprise.

I declare that I am aware that the intended purpose of performing the forenamed procedure may not be achieved, even though the physician and his/her team adopt the best techniques and employ all of the scientific means and resources available.

I am also aware that the procedure involves risks, and I have received all the pertinent information regarding possible complications due to known and unknown causes, including death, stroke, myocardial infarction, cardiac arrhythmia, acute pulmonary edema, anaphylactic shock, varying types and degrees of infection, allergies and/or reactions to contrast, bleedings, hematomas, renal insufficiency, vascular and hemodynamic complications, perforation of cardiac chambers or vessels, and loss of limbs and/or their function, in addition to the risks inherent in anesthesia and the use of diverse instruments and equipment itself. It has also been explained to me that these adverse reactions and infrequent, occurring in less than 5% of cases, but they may be aggravated when associated with other patient personal factors, which include underlying diseases, previous heart surgery, prior history of allergies, uncontrolled arterial hypertension, tobacco use, alcoholism, diabetes, obesity, renal insufficiency, cerebrovascular disease, prolonged hospitalization, liver failure, heart disease, atherosclerotic disease, cancer, severe malnutrition, and advanced age, which are the most common.

It has also been explained to me that the catheters and prostheses used are subjected to previous tests, but that they may present defects or even fracture, causing adverse reactions and varying types and degrees of injury, including the possibility of requiring surgery to remove them. These risks, however, are less than 1%.

It has been reiterated to me that no guarantees or assurances are given with respect to the results expected from the proposed procedure. I am aware that, in executing the proposed procedure, the hemodynamicist and his/her team will be present, and it will be possible to solicit the presence of other specialists, as well as observers from the manufacturer of the equipment and material used. My signature at the end of this consent form authorizes the participation of these professionals and grants them full access to records and medical information related to my case. I have also been informed that, should it be necessary, bed restraint may be performed, as prescribed by the physician, and that I will be under the surveillance of the nursing staff.

I further declare that I have had the opportunity to read this document carefully and also to listen attentively to the physician’s reading of all the information presented herein, and I was able to clarify all of my doubts regarding the risks involved and the possible consequences of not performing the suggested treatment, as well as the possible existence of alternative procedures, and my doubts have been satisfactorily addressed and answered. I am aware that there is a possibility of alteration of the original procedure over the course of the aforementioned procedure and/or new procedure or surgery for the sake of continuity of treatment in a different region from the one originally designated, which, if applicable, will be evaluated, decided upon, and performed, by the medical professional in charge, with which I now agree and hereby authorize.

All my questions having been answered and having understood the procedure in and of itself, as well as its real repercussions, I sign this CONSENT FORM, in the presence of Dr(s). ....................................................... and ........................................................., Who, in this act, represents the Hemodynamic Medical Service, together with 2 (two) witnesses, expressly and voluntarily declaring my authorization for the procedure of transcatheter aortic valve implantation (TAVI), consenting, furthermore, to the performance of the proposed procedure as well as its preparatory acts; the physicians are hereby authorized to performed additional procedures and therapeutic interventions that may become necessary due to unforeseen conditions which may occur during the course of the procedure, remaining always at the discretion and judgment of the attending physician. Finally, I consent to the eventual possibility of a blood transfusion, should this procedure be directly linked to the treatment mentioned in this consent and authorization form or resulting from it.

If, during or immediately after the performance of the procedure which I am to undergo, I am not in full possession of my physical and/or mental abilities, I authorize my legal representative ................................................................................................................................, nationality ...................................................., marital status ......................................................., profession ....................................................., identity document .................................................., issued by ................................., CPF ..............................................................., resident of ......................................................................................................................................, city ................................................................, state ................., date of birth ........../........../.........., parents’ names........................................................................................................................................., relationship to patient ............................................................, to make decisions in my name.

In this manner, I offer my full and free consent, and I authorize the performance of the procedure of transcatheter aortic valve implantation (TAVI), explained herein, to be administered by the signing physician and his/her related medical team.

In conclusion, I certify, that I have signed two copies of the present Form, one of which has been given to me.

**Table:**

Center Location: ..........................................	
Date: ........../........../..........	
Patient's Name: ......................................................	Signature: ..................................................
	
Representative: ............................................	Signature: ...................................................
	
Physician's Name: ...................................................	CRM nº.: ..........................
Signature: ..................................................	
Dr.(a) ...............................................	CRM nº.: ..........................
Signature: ..................................................	
	
Witnesses:	
Name: ........................................................ Name: ........................................................	

## Annex 8

**FREE AND INFORMED CONSENT AND AUTHORIZATION FORM FOR PERFORMANCE OF THE PROCEDURE OF ALCOHOL SEPTAL ABLATION**

By the present Consent and Authorization Form, I, ........................................................................................................, nationality ................................................, a legal adult and able, marital status .........................................................., profession ....................................................., identity document ......................................................., issued by ......................................., CPF ...................................................., resident of ..........................................................................................................................................., city .............................................................., state ....................., date of birth ........../........../.........., parents’ names ........................................................................................................................................., hereby declare that I have received from the Hemodynamics Service, here represented by the physician fully identified below, explanations and warnings concerning the procedure solicited by my clinical physician and that the discussion regarding the nature and extent of the actions necessary for its execution is registered below.

I further declare that it has been explained to me that the procedure alcohol septal ablation will be performed in the hemodynamics laboratory of this hospital institution (corporate name ........................................................................................................................., CNPJ/MF no. ...................................headquarters....................................................................) and that it consists of punctures in the patient’s skin in order to introduce special catheters, using iodized contrast, with the administration of local anesthesia, sedation, or general anesthesia, at the attending physician’s discretion.

The following text has been read and explained to me in more accessible language by the signing physician: the aim of this procedure is to reduce significant muscular thickening in the interventricular septum (which separates the left and right ventricles) by means of alcoholization of the septal artery accessed by puncture of an artery in the inguinal region. Subsequently, a catheter is selectively placed in the left coronary artery. Through this catheter a very small guidewire is positioned in the first septal branch of the anterior descending coronary artery. This guidewire will allow for the correct positioning of a catheter specifically dedicated to this purpose, which has a small balloon on its end. This balloon will be inflated, closing the septal branch in its proximal portion and, then, one to two milliliters of absolute alcohol or other liquid substances or mechanical microdevices will be administered through the balloon catheter to the distal end of the septal branch. The alcohol or other agents will provoke direct damage to the thickened muscle of the interventricular septum, which, during the course of weeks or even months, will reduce in volume. From that moment onward, the mitral valve will function better and the pressure in the left ventricle will be reduced, which will result in improvements to symptoms of cardiac insufficiency.

I declare that, in the manner explained to me by the physician, I am perfectly able to understand what the procedure which I am to undergo will comprise.

I am also aware that it may take weeks or months for the interventricular septum to become reduced and for me to observe the complete benefit of this procedure. A temporary pacemaker lead may be placed in the right ventricle through the venous access in the inguinal region. Echocardiogram may be performed during the procedure in accordance with the medical team’s indications.

I declare that I am aware that the intended purpose of performing the forenamed procedure may not be achieved, even though the physician and his/her team adopt the best techniques and employ all of the scientific means and resources available.

I am also aware that the procedure involves risks, and I have received all the pertinent information regarding possible complications due to known and unknown causes, including death, stroke, myocardial infarction, cardiac arrhythmia, acute pulmonary edema, anaphylactic shock, varying types and degrees of infection, allergies and/or reactions to contrast, bleedings, hematomas, renal insufficiency, vascular and hemodynamic complications, perforation of cardiac chambers or vessels, and loss of limbs and/or their function, in addition to the risks inherent in anesthesia and the use of diverse instruments and equipment itself. It has also been explained to me that these adverse reactions and infrequent, occurring in approximately 2% to 8% of cases, but they may be aggravated when associated with other patient personal factors, which include underlying diseases, previous heart surgery, prior history of allergies, uncontrolled arterial hypertension, tobacco use, alcoholism, diabetes, obesity, renal insufficiency, cerebrovascular disease, prolonged hospitalization, liver failure, heart disease, atherosclerotic disease, cancer, severe malnutrition, and advanced age, which are the most common.

It has also been explained to me that the catheters and prostheses used are subjected to previous tests, but that they may present defects or even fracture, causing adverse reactions and varying types and degrees of injury, including the possibility of requiring surgery to remove them.

It has been reiterated to me that no guarantees or assurances are given with respect to the results expected from the proposed procedure. I am aware that, in executing the proposed procedure, the hemodynamicist and his/her team will be present, and it will be possible to solicit the presence of other specialists, as well as observers from the manufacturer of the equipment and material used. My signature at the end of this consent form authorizes the participation of these professionals and grants them full access to records and medical information related to my case. I have also been informed that, should it be necessary, bed restraint may be performed, as prescribed by the physician, and that I will be under the surveillance of the nursing staff.

I further declare that I have had the opportunity to read this document carefully and also to listen attentively to the physician’s reading of all the information presented herein, and I was able to clarify all of my doubts regarding the risks involved and the possible consequences of not performing the suggested treatment, as well as the possible existence of alternative procedures, and my doubts have been satisfactorily addressed and answered. I am aware that there is a possibility of alteration of the original procedure over the course of the aforementioned procedure and/or new procedure or surgery for the sake of continuity of treatment in a different region from the one originally designated, which, if applicable, will be evaluated, decided upon, and performed, by the medical professional in charge, with which I now agree and hereby authorize.

All my questions having been answered and having understood the procedure in and of itself, as well as its real repercussions, I sign this CONSENT FORM, in the presence of Dr. ................................................................................................................, who, in this act, represents the Hemodynamic Medical Service, together with 2 (two) witnesses, expressly and voluntarily declaring my authorization for the procedure of alcohol septal ablation, consenting, furthermore, to the performance of the proposed procedure as well as its preparatory acts; the physicians are hereby authorized to performed additional procedures and therapeutic interventions that may become necessary due to unforeseen conditions which may occur during the course of the procedure, remaining always at the discretion and judgment of the attending physician. Finally, I consent to the eventual possibility of a blood transfusion, should this procedure be directly linked to the treatment mentioned in this consent and authorization form or resulting from it.

If, during or immediately after the performance of the procedure which I am to undergo, I am not in full possession of my physical and/or mental abilities, I authorize my legal representative ................................................................................................................................, nationality ...................................................., marital status ......................................................., profession ....................................................., identity document .................................................., issued by ................................., CPF ..............................................................., resident of ......................................................................................................................................, city ................................................................, state ................., date of birth ........../........../.........., parents’ names........................................................................................................................................., relationship to patient ............................................................, to make decisions in my name.

In this manner, I offer my full and free consent, and I authorize the performance of the procedure of alcohol septal ablation, explained herein, to be administered by the signing physician and his/her related medical team.

In conclusion, I certify, that I have signed two copies of the present Form, one of which has been given to me.

**Table:**

Center Location: ..........................................	
Date: ........../........../..........	
Patient's Name: ......................................................	Signature: ..................................................
	
Representative: .........................	Signature: ...................................................
	
Physician's Name: ...................................................	CRM nº.: ..........................
Signature: ..................................................	
	
Witnesses:	
Name: ........................................................ Name: ........................................................	

## Annex 9

**FREE AND INFORMED CONSENT AND AUTHORIZATION FORM FOR PERFORMANCE OF THE PROCEDURE OF PATENT FORAMEN OVALE OCCLUSION OR CLOSURE**

By the present Consent and Authorization Form, I, .................................................................................................................., nationality ..............................................., a legal adult and able, marital status .........................................................., profession ....................................................., identity document ......................................................., issued by ........................................, CPF ...................................................., resident of ..........................................................................................................................................., city ................................................, state ....................., date of birth ........../........../.........., parents’ names ........................................................................................................................................., hereby declare that I have received from the Hemodynamics Service, here represented by the physician fully identified below, explanations and warnings concerning the procedure solicited by my clinical physician and that the discussion regarding the nature and extent of the actions necessary for its execution is registered below.

I further declare that it has been explained to me that the procedure patent foramen ovale occlusion or closure will be performed in the hemodynamics laboratory of this hospital institution (corporate name .................................................................................................................., CNPJ/MF no. ...................................headquarters....................................................................) and that it consists of punctures in the patient’s skin in order to introduce special catheters, prostheses and/or devices, using iodized contrast, with the administration of local anesthesia, sedation, or general anesthesia, at the attending physician’s discretion.

The following text has been read and explained to me in more accessible language by the signing physician: the aim of this procedure is to repair abnormal communication, resulting from the persistence of a small opening or orifice, known as patent foramen ovale (PFO), located between the right and left atria. The procedure is performed by means of a specific catheter, introduced in the inguinal region or groin, and it has been recommended by a multidisciplinary team of specialists for patients who have clinical and anatomical characteristics favorable to this approach. The placement of a device similar to an “umbrella,” known as a prosthesis for PFO closure, occurs by means of the insertion of a catheter in a vein in the inguinal region to the left atrium of the heart, through the PFO. The entire procedure is guided by X-ray and mainly by transesophageal echocardiography, which will aid the medical team in the correct positioning and safe release of the prosthesis in the PFO. Once the device has been successfully released, the catheter will be removed and maneuvers will be performed to contain eventual bleedings in the puncture site, and the procedure will subsequently be concluded.

I declare that, in the manner explained to me by the physician, I am perfectly able to understand what the procedure which I am to undergo will comprise.

I declare that I am aware that the intended purpose of performing the forenamed procedure may not be achieved, even though the physician and his/her team adopt the best techniques and employ all of the scientific means and resources available.

I am also aware that the procedure involves risks, and I have received all the pertinent information regarding possible complications due to known and unknown causes, including death, stroke, myocardial infarction, cardiac arrhythmia, acute pulmonary edema, anaphylactic shock, varying types and degrees of infection, allergies and/or reactions to contrast, bleedings, hematomas, renal insufficiency, vascular and hemodynamic complications, perforation of cardiac chambers or vessels, and loss of limbs and/or their function, in addition to the risks inherent in anesthesia and the use of diverse instruments and equipment itself. It has also been explained to me that these adverse reactions and infrequent, occurring in less than 2% of cases, but they may be aggravated when associated with other patient personal factors, which include underlying diseases, previous heart surgery, prior history of allergies, uncontrolled arterial hypertension, tobacco use, alcoholism, diabetes, obesity, renal insufficiency, cerebrovascular disease, prolonged hospitalization, liver failure, heart disease, atherosclerotic disease, cancer, severe malnutrition, and advanced age, which are the most common.

It has also been explained to me that the catheters and prostheses used are subjected to previous tests, but that they may present defects or even fracture, causing adverse reactions and varying types and degrees of injury, including the possibility of requiring surgery to remove them.

It has been reiterated to me that no guarantees or assurances are given with respect to the results expected from the proposed procedure. I am aware that, in executing the proposed procedure, the hemodynamicist and his/her team will be present, and it will be possible to solicit the presence of other specialists, as well as observers from the manufacturer of the equipment and material used. My signature at the end of this consent form authorizes the participation of these professionals and grants them full access to records and medical information related to my case. I have also been informed that, should it be necessary, bed restraint may be performed, as prescribed by the physician, and that I will be under the surveillance of the nursing staff.

I further declare that I have had the opportunity to read this document carefully and also to listen attentively to the physician’s reading of all the information presented herein, and I was able to clarify all of my doubts regarding the risks involved and the possible consequences of not performing the suggested treatment, as well as the possible existence of alternative procedures, and my doubts have been satisfactorily addressed and answered. I am aware that there is a possibility of alteration of the original procedure over the course of the aforementioned procedure and/or new procedure or surgery for the sake of continuity of treatment in a different region from the one originally designated, which, if applicable, will be evaluated, decided upon, and performed, by the medical professional in charge, with which I now agree and hereby authorize.

All my questions having been answered and having understood the procedure in and of itself, as well as its real repercussions, I sign this CONSENT FORM, in the presence of Dr. ................................................................................................................, who, in this act, represents the Hemodynamic Medical Service, together with 2 (two) witnesses, expressly and voluntarily declaring my authorization for the procedure of patent foramen ovale occlusion or closure, consenting, furthermore, to the performance of the proposed procedure as well as its preparatory acts; the physicians are hereby authorized to performed additional procedures and therapeutic interventions that may become necessary due to unforeseen conditions which may occur during the course of the procedure, remaining always at the discretion and judgment of the attending physician. Finally, I consent to the eventual possibility of a blood transfusion, should this procedure be directly linked to the treatment mentioned in this consent and authorization form or resulting from it.

If, during or immediately after the performance of the procedure which I am to undergo, I am not in full possession of my physical and/or mental abilities, I authorize my legal representative ................................................................................................................................, nationality ...................................................., marital status ......................................................., profession ....................................................., identity document .................................................., issued by ................................., CPF ..............................................................., resident of ......................................................................................................................................, city ................................................................, state ................., date of birth ........../........../.........., parents’ names........................................................................................................................................., relationship to patient ............................................................, to make decisions in my name.

In this manner, I offer my full and free consent, and I authorize the performance of the procedure of patent foramen ovale occlusion or closure, explained herein, to be administered by the signing physician and his/her related medical team.

In conclusion, I certify, that I have signed two copies of the present Form, one of which has been given to me.

**Table:**

Center Location: ..........................................
Date: ........../........../..........
Patient's Name: ...................................................... Signature: ..................................................

## Annex 10

**FREE AND INFORMED CONSENT AND AUTHORIZATION FORM FOR PERFORMANCE OF THE PROCEDURE OF INTERATRIAL COMMUNICATION CLOSURE OR OCCLUSION**

By the present Consent and Authorization Form, I, ........................................................................................................, nationality ................................................, a legal adult and able, marital status .........................................................., profession ....................................................., identity document ......................................................., issued by ......................................., CPF ...................................................., resident of ..........................................................................................................................................., city .............................................................., state ....................., date of birth ........../........../.........., parents’ names ........................................................................................................................................., hereby declare that I have received from the Hemodynamics Service, here represented by the physician fully identified below, explanations and warnings concerning the procedure solicited by my clinical physician and that the discussion regarding the nature and extent of the actions necessary for its execution is registered below.

I further declare that it has been explained to me that the procedure interatrial communication closure or occlusion will be performed in the hemodynamics laboratory of this hospital institution (corporate name .............................................................................................., CNPJ/MF no. ...................................headquarters....................................................................) and that it consists of a puncture in the skin of the patient in order to introduce special catheters, using iodized contrast, with the administration of local anesthesia, sedation, or general anesthesia, at the attending physician’s discretion.

The following text has been read and explained to me in more accessible language by the signing physician: the aim of this procedure is to repair abnormal communication of a small opening (orifice) located in the septum that separates two cavities of the heart (right and left atria), known as interatrial communication (IAC). The procedure consists of the introduction of a specific catheter in the inguinal region or groin. This procedure has been recommended by a multidisciplinary team of specialists for patients who have clinical and anatomical characteristics favorable to this approach. The placement of a device similar to an “umbrella” or a spring, known as a prosthesis for IAC closure, occurs by means of the insertion of a catheter in a vein in the inguinal region to the left atrium of the heart, through the IAC. The entire procedure is guided by X-ray and mainly by transesophageal echocardiography, which will aid the medical team in the correct positioning and safe release of the prosthesis in the IAC. Once the device has been successfully released, the catheter will be removed and maneuvers will be performed to contain eventual bleedings in the puncture site, and the procedure will subsequently be concluded.

I declare that, in the manner explained to me by the physician, I am perfectly able to understand what the procedure which I am to undergo will comprise.

I declare that I am aware that the intended purpose of performing the forenamed procedure may not be achieved, even though the physician and his/her team adopt the best techniques and employ all of the scientific means and resources available.

I am also aware that the procedure involves risks, and I have received all the pertinent information regarding possible complications due to known and unknown causes, including death, stroke, myocardial infarction, cardiac arrhythmia, acute pulmonary edema, anaphylactic shock, varying types and degrees of infection, allergies and/or reactions to contrast, bleedings, hematomas, renal insufficiency, vascular and hemodynamic complications, perforation of cardiac chambers or vessels, and loss of limbs and/or their function, in addition to the risks inherent in anesthesia and the use of diverse instruments and equipment itself. It has also been explained to me that these adverse reactions and infrequent, occurring in less than 2% of cases, but they may be aggravated when associated with other patient personal factors, which include underlying diseases, previous heart surgery, prior history of allergies, uncontrolled arterial hypertension, tobacco use, alcoholism, diabetes, obesity, renal insufficiency, cerebrovascular disease, prolonged hospitalization, liver failure, heart disease, atherosclerotic disease, cancer, severe malnutrition, and advanced age, which are the most common.

It has also been explained to me that the catheters and prostheses used are subjected to previous tests, but that they may present defects or even fracture, causing adverse reactions and varying types and degrees of injury, including the possibility of requiring surgery to remove them.

It has been reiterated to me that no guarantees or assurances are given with respect to the results expected from the proposed procedure. I am aware that, in executing the proposed procedure, the hemodynamicist and his/her team will be present, and it will be possible to solicit the presence of other specialists, as well as observers from the manufacturer of the equipment and material used. My signature at the end of this consent form authorizes the participation of these professionals and grants them full access to records and medical information related to my case. I have also been informed that, should it be necessary, bed restraint may be performed, as prescribed by the physician, and that I will be under the surveillance of the nursing staff.

I further declare that I have had the opportunity to read this document carefully and also to listen attentively to the physician’s reading of all the information presented herein, and I was able to clarify all of my doubts regarding the risks involved and the possible consequences of not performing the suggested treatment, as well as the possible existence of alternative procedures, and my doubts have been satisfactorily addressed and answered. I am aware that there is a possibility of alteration of the original procedure over the course of the aforementioned procedure and/or new procedure or surgery for the sake of continuity of treatment in a different region from the one originally designated, which, if applicable, will be evaluated, decided upon, and performed, by the medical professional in charge, with which I now agree and hereby authorize.

All my questions having been answered and having understood the procedure in and of itself, as well as its real repercussions, I sign this CONSENT FORM, in the presence of Dr. ................................................................................................................, who, in this act, represents the Hemodynamic Medical Service, together with 2 (two) witnesses, expressly and voluntarily declaring my authorization for the procedure of interatrial communication closure or occlusion, consenting, furthermore, to the performance of the proposed procedure as well as its preparatory acts; the physicians are hereby authorized to performed additional procedures and therapeutic interventions that may become necessary due to unforeseen conditions which may occur during the course of the procedure, remaining always at the discretion and judgment of the attending physician. Finally, I consent to the eventual possibility of a blood transfusion, should this procedure be directly linked to the treatment mentioned in this consent and authorization form or resulting from it.

If, during or immediately after the performance of the procedure which I am to undergo, I am not in full possession of my physical and/or mental abilities, I authorize my legal representative ................................................................................................................................, nationality ...................................................., marital status ......................................................., profession ....................................................., identity document .................................................., issued by ................................., CPF ..............................................................., resident of ......................................................................................................................................, city ................................................................, state ................., date of birth ........../........../.........., parents’ names........................................................................................................................................., relationship to patient ............................................................, to make decisions in my name.

In this manner, I offer my full and free consent, and I authorize the performance of the procedure of interatrial communication closure or occlusion, explained herein, to be administered by the signing physician and his/her related medical team.

In conclusion, I certify, that I have signed two copies of the present Form, one of which has been given to me.

**Table:**

Center Location: ..........................................	
Date: ........../........../..........	
Patient's Name: ......................................................	Signature: ..................................................
	
Representative: ............................................	Signature: ...................................................
	
Physician's Name: ...................................................	CRM nº.: ..........................
Signature: ..................................................	
	
Witnesses:	
Name: ........................................................ Name: ........................................................	

## Annex 11

**FREE AND INFORMED CONSENT AND AUTHORIZATION FORM FOR PERFORMANCE OF THE PROCEDURE OF TRANSCATHETER INTERVENTRICULAR COMMUNICATION CLOSURE OR OCCLUSION**

By the present Consent and Authorization Form, I, ........................................................................................................, nationality ................................................, a legal adult and able, marital status .........................................................., profession ....................................................., identity document ......................................................., issued by ......................................., CPF ...................................................., resident of ..........................................................................................................................................., city .............................................................., state ....................., date of birth ........../........../.........., parents’ names ........................................................................................................................................., hereby declare that I have received from the Hemodynamics Service, here represented by the physician fully identified below, explanations and warnings concerning the procedure solicited by my clinical physician and that the discussion regarding the nature and extent of the actions necessary for its execution is registered below.

I further declare that it has been explained to me that the procedure transcatheter interventricular communication closure or occlusion will be performed in the hemodynamics laboratory of this hospital institution (corporate name ......................................................................................., CNPJ/MF no. ...................................headquarters....................................................................) and that it consists of a puncture in the skin of the patient in order to introduce special catheters, using iodized contrast, with the administration of local anesthesia, sedation, or general anesthesia, at the attending physician’s discretion.

The following text has been read and explained to me in more accessible language by the signing physician: the aim of this procedure is to repair abnormal communication resulting from a small opening (orifice) located in the septum that separates two cavities of the heart, the right and left ventricles, known as interventricular communication (IVC). The procedure consists of the introduction of a specific catheter in the inguinal region or groin. This procedure has been recommended by a multidisciplinary team of specialists for patients who have clinical and anatomical characteristics favorable to this approach. The placement of a device similar to an “umbrella” or a spring, known as a prosthesis for IVC closure, occurs by means of the insertion of a catheter in a artery in the inguinal region to the left ventricle of the heart. The entire procedure is guided by X-ray and mainly by transesophageal echocardiography, which will aid the medical team in the correct positioning and safe release of the prosthesis in the IVC. Once the device has been successfully released, the catheter will be removed and maneuvers will be performed to contain eventual bleedings in the puncture site, and the procedure will be concluded.

I declare that, in the manner explained to me by the physician, I am perfectly able to understand what the procedure which I am to undergo will comprise.

I declare that I am aware that the intended purpose of performing the forenamed procedure may not be achieved, even though the physician and his/her team adopt the best techniques and employ all of the scientific means and resources available.

I am also aware that the procedure involves risks, and I have received all the pertinent information regarding possible complications due to known and unknown causes, including death, stroke, myocardial infarction, cardiac arrhythmia, acute pulmonary edema, anaphylactic shock, varying types and degrees of infection, allergies and/or reactions to contrast, bleedings, hematomas, renal insufficiency, vascular and hemodynamic complications, perforation of cardiac chambers or vessels, and loss of limbs and/or their function, in addition to the risks inherent in anesthesia and the use of diverse instruments and equipment itself. It has also been explained to me that these adverse reactions and infrequent, occurring in less than 2% of cases, but they may be aggravated when associated with other patient personal factors, which include underlying diseases, previous heart surgery, prior history of allergies, uncontrolled arterial hypertension, tobacco use, alcoholism, diabetes, obesity, renal insufficiency, cerebrovascular disease, prolonged hospitalization, liver failure, heart disease, atherosclerotic disease, cancer, severe malnutrition, and advanced age, which are the most common.

It has also been explained to me that the catheters and prostheses used are subjected to previous tests, but that they may present defects or even fracture, causing adverse reactions and varying types and degrees of injury, including the possibility of requiring surgery to remove them.

It has been reiterated to me that no guarantees or assurances are given with respect to the results expected from the proposed procedure. I am aware that, in executing the proposed procedure, the hemodynamicist and his/her team will be present, and it will be possible to solicit the presence of other specialists, as well as observers from the manufacturer of the equipment and material used. My signature at the end of this consent form authorizes the participation of these professionals and grants them full access to records and medical information related to my case. I have also been informed that, should it be necessary, bed restraint may be performed, as prescribed by the physician, and that I will be under the surveillance of the nursing staff.

I further declare that I have had the opportunity to read this document carefully and also to listen attentively to the physician’s reading of all the information presented herein, and I was able to clarify all of my doubts regarding the risks involved and the possible consequences of not performing the suggested treatment, as well as the possible existence of alternative procedures, and my doubts have been satisfactorily addressed and answered. I am aware that there is a possibility of alteration of the original procedure over the course of the aforementioned procedure and/or new procedure or surgery for the sake of continuity of treatment in a different region from the one originally designated, which, if applicable, will be evaluated, decided upon, and performed, by the medical professional in charge, with which I now agree and hereby authorize.

All my questions having been answered and having understood the procedure in and of itself, as well as its real repercussions, I sign this CONSENT FORM, in the presence of Dr. ................................................................................................................, who, in this act, represents the Hemodynamic Medical Service, together with 2 (two) witnesses, expressly and voluntarily declaring my authorization for the procedure of transcatheter interventricular communication closure or occlusion, consenting, furthermore, to the performance of the proposed procedure as well as its preparatory acts; the physicians are hereby authorized to performed additional procedures and therapeutic interventions that may become necessary due to unforeseen conditions which may occur during the course of the procedure, remaining always at the discretion and judgment of the attending physician. Finally, I consent to the eventual possibility of a blood transfusion, should this procedure be directly linked to the treatment mentioned in this consent and authorization form or resulting from it.

If, during or immediately after the performance of the procedure which I am to undergo, I am not in full possession of my physical and/or mental abilities, I authorize my legal representative ................................................................................................................................, nationality ...................................................., marital status ......................................................., profession ....................................................., identity document .................................................., issued by ................................., CPF ..............................................................., resident of ......................................................................................................................................, city ................................................................, state ................., date of birth ........../........../.........., parents’ names........................................................................................................................................., relationship to patient ............................................................, to make decisions in my name.

In this manner, I offer my full and free consent, and I authorize the performance of the procedure of transcatheter interventricular communication closure or occlusion, explained herein, to be administered by the signing physician and his/her related medical team.

In conclusion, I certify, that I have signed two copies of the present Form, one of which has been given to me.

**Table:**

Center Location: ..........................................	
Date: ........../........../..........	
Patient's Name: ......................................................	Signature: ..................................................
	
Representative: ............................................	Signature: ...................................................
	
Physician's Name: ...................................................	CRM nº.: ..........................
Signature: ..................................................	
	
Witnesses:	
Name: ........................................................ Name: ........................................................	

## Annex 12

**FREE AND INFORMED CONSENT AND AUTHORIZATION FORM FOR PERFORMANCE OF THE PROCEDURE OF PATENT DUCTUS ARTERIOSUS CLOSURE OR OCCLUSION**

By the present Consent and Authorization Form, I, ........................................................................................................, nationality ................................................, a legal adult and able, marital status .........................................................., profession ....................................................., identity document ......................................................., issued by ......................................., CPF ...................................................., resident of ..........................................................................................................................................., city .............................................................., state ....................., date of birth ........../........../.........., parents’ names ........................................................................................................................................., hereby declare that I have received from the Hemodynamics Service, here represented by the physician fully identified below, explanations and warnings concerning the procedure solicited by my clinical physician and that the discussion regarding the nature and extent of the actions necessary for its execution is registered below.

I further declare that it has been explained to me that the procedure patent ductus arteriosus closure or occlusion will be performed in the hemodynamics laboratory of this hospital institution (corporate name ........................................................................................................., CNPJ/MF no. ...................................headquarters....................................................................) and that it consists of a puncture in the skin of the patient in order to introduce special catheters, using iodized contrast, with the administration of local anesthesia, sedation, or general anesthesia, at the attending physician’s discretion.

The following text has been read and explained to me in more accessible language by the signing physician: the aim of this procedure is to repair abnormal communication through a small channel or tube between two important arteries in the heart, the aorta and the pulmonary artery, known as patent ductus arteriosus (PDA). The procedure consists of the introduction of specific guidewires and catheters in the right and/or left inguinal region in order to correct this congenital defect. This procedure has been recommended by a multidisciplinary team of specialists for patients who have clinical and anatomical characteristics favorable to this approach. This communication is closed by means of devices similar to plugs (occluders) or even with coils (springs). The entire procedure is guided by X-ray, which will aid the medical team in the correct positioning and safe release of the prosthesis in the PDA. Once the device has been successfully released, the catheter will be removed and maneuvers will be performed to contain eventual bleedings in the puncture site, and the procedure will subsequently be concluded.

I declare that, in the manner explained to me by the physician, I am perfectly able to understand what the procedure which I am to undergo will comprise.

I declare that I am aware that the intended purpose of performing the forenamed procedure may not be achieved, even though the physician and his/her team adopt the best techniques and employ all of the scientific means and resources available.

I am also aware that the procedure involves risks, and I have received all the pertinent information regarding possible complications due to known and unknown causes, including death, stroke, myocardial infarction, cardiac arrhythmia, acute pulmonary edema, anaphylactic shock, varying types and degrees of infection, allergies and/or reactions to contrast, bleedings, hematomas, renal insufficiency, vascular and hemodynamic complications, perforation of cardiac chambers or vessels, and loss of limbs and/or their function, in addition to the risks inherent in anesthesia and the use of diverse instruments and equipment itself. It has also been explained to me that these adverse reactions and infrequent, occurring in less than 2% of cases, but they may be aggravated when associated with other patient personal factors, which include underlying diseases, previous heart surgery, prior history of allergies, uncontrolled arterial hypertension, tobacco use, alcoholism, diabetes, obesity, renal insufficiency, cerebrovascular disease, prolonged hospitalization, liver failure, heart disease, atherosclerotic disease, cancer, severe malnutrition, and advanced age, which are the most common.

It has also been explained to me that the catheters and prostheses used are subjected to previous tests, but that they may present defects or even fracture, causing adverse reactions and varying types and degrees of injury, including the possibility of requiring surgery to remove them.

It has been reiterated to me that no guarantees or assurances are given with respect to the results expected from the proposed procedure. I am aware that, in executing the proposed procedure, the hemodynamicist and his/her team will be present, and it will be possible to solicit the presence of other specialists, as well as observers from the manufacturer of the equipment and material used. My signature at the end of this consent form authorizes the participation of these professionals and grants them full access to records and medical information related to my case. I have also been informed that, should it be necessary, bed restraint may be performed, as prescribed by the physician, and that I will be under the surveillance of the nursing staff.

I further declare that I have had the opportunity to read this document carefully and also to listen attentively to the physician’s reading of all the information presented herein, and I was able to clarify all of my doubts regarding the risks involved and the possible consequences of not performing the suggested treatment, as well as the possible existence of alternative procedures, and my doubts have been satisfactorily addressed and answered. I am aware that there is a possibility of alteration of the original procedure over the course of the aforementioned procedure and/or new procedure or surgery for the sake of continuity of treatment in a different region from the one originally designated, which, if applicable, will be evaluated, decided upon, and performed, by the medical professional in charge, with which I now agree and hereby authorize.

All my questions having been answered and having understood the procedure in and of itself, as well as its real repercussions, I sign this CONSENT FORM, in the presence of Dr. ................................................................................................................, who, in this act, represents the Hemodynamic Medical Service, together with 2 (two) witnesses, expressly and voluntarily declaring my authorization for the procedure of patent ductus arteriosus closure or occlusion, consenting, furthermore, to the performance of the proposed procedure as well as its preparatory acts; the physicians are hereby authorized to performed additional procedures and therapeutic interventions that may become necessary due to unforeseen conditions which may occur during the course of the procedure, remaining always at the discretion and judgment of the attending physician. Finally, I consent to the eventual possibility of a blood transfusion, should this procedure be directly linked to the treatment mentioned in this consent and authorization form or resulting from it.

If, during or immediately after the performance of the procedure which I am to undergo, I am not in full possession of my physical and/or mental abilities, I authorize my legal representative ................................................................................................................................, nationality ...................................................., marital status ......................................................., profession ....................................................., identity document .................................................., issued by ................................., CPF ..............................................................., resident of ......................................................................................................................................, city ................................................................, state ................., date of birth ........../........../.........., parents’ names........................................................................................................................................., relationship to patient ............................................................, to make decisions in my name.

In this manner, I offer my full and free consent, and I authorize the performance of the procedure of patent ductus arteriosus closure or occlusion, explained herein, to be administered by the signing physician and his/her related medical team.

In conclusion, I certify, that I have signed two copies of the present Form, one of which has been given to me.

**Table:**

Center Location: ..........................................	
Date: ........../........../..........	
Patient's Name: ......................................................	Signature: ..................................................
	
Representative: ............................................	Signature: ...................................................
	
Physician's Name: ...................................................	CRM nº.: ..........................
Signature: ..................................................	
	
Witnesses:	
Name: ........................................................ Name: ........................................................	

## Annex 13

**FREE AND INFORMED CONSENT AND AUTHORIZATION FORM FOR PERFORMANCE OF THE PROCEDURE OF ANGIOPLASTY WITH CAROTID STENT IMPLANTATION**

By the present Consent and Authorization Form, I, ........................................................................................................, nationality ................................................, a legal adult and able, marital status .........................................................., profession ....................................................., identity document ......................................................., issued by ......................................., CPF ...................................................., resident of ..........................................................................................................................................., city .............................................................., state ....................., date of birth ........../........../.........., parents’ names ........................................................................................................................................., hereby declare that I have received from the Hemodynamics Service, here represented by the physician fully identified below, explanations and warnings concerning the procedure solicited by my clinical physician and that the discussion regarding the nature and extent of the actions necessary for its execution is registered below.

I further declare that it has been explained to me that the procedure angioplasty with carotid stent implantation will be performed in the hemodynamics laboratory of this hospital institution (corporate name .............................................................................................., CNPJ/MF no. ...................................headquarters....................................................................) and that it consists of a puncture in the skin of the patient in order to introduce special catheters, using iodized contrast, with the administration of local anesthesia, sedation, or general anesthesia, at the attending physician’s discretion.

The following text has been read and explained to me in more accessible language by the signing physician: the aim of this procedure is to treat obstructions or blocks in carotid arteries by means of the implantation of a metallic mesh called a stent, via percutaneous access. The procedure is performed by means of a puncture in the femoral artery in the inguinal region, into which a catheter is introduced close to the diseased carotid artery. X-ray emitting equipment and iodized contrast are used to identify the block in the artery. A very small guidewire will cross the obstruction, allowing for positioning of a small device to filter any particle or clot that may come loose from the block being treated. Subsequently, a stent will be positioned and released in the site. Following a few tests, a catheter with a balloon on its end will be inflated inside the stent to improve the final result. Finally, the catheter, the filter, and the guidewire will be removed, and cerebral arteriography will be performed as final angiographic control.

I declare that, in the manner explained to me by the physician, I am perfectly able to understand what the procedure which I am to undergo will comprise.

I declare that I am aware that the intended purpose of performing the forenamed procedure may not be achieved, even though the physician and his/her team adopt the best techniques and employ all of the scientific means and resources available.

I am also aware that the procedure involves risks, and I have received all the pertinent information regarding possible complications due to known and unknown causes, including death, stroke, myocardial infarction, cardiac arrhythmia, acute pulmonary edema, anaphylactic shock, varying types and degrees of infection, allergies and/or reactions to contrast, bleedings, hematomas, renal insufficiency, vascular and hemodynamic complications, perforation of cardiac chambers or vessels, and loss of limbs and/or their function, in addition to the risks inherent in anesthesia and the use of diverse instruments and equipment itself. It has also been explained to me that these adverse reactions and infrequent, occurring in less than 2% of cases, but they may be aggravated when associated with other patient personal factors, which include underlying diseases, previous heart surgery, prior history of allergies, uncontrolled arterial hypertension, tobacco use, alcoholism, diabetes, obesity, renal insufficiency, cerebrovascular disease, prolonged hospitalization, liver failure, heart disease, atherosclerotic disease, cancer, severe malnutrition, and advanced age, which are the most common.

It has also been explained to me that the catheters and prostheses used are subjected to previous tests, but that they may present defects or even fracture, causing adverse reactions and varying types and degrees of injury, including the possibility of requiring surgery to remove them.

It has been reiterated to me that no guarantees or assurances are given with respect to the results expected from the proposed procedure. I am aware that, in executing the proposed procedure, the hemodynamicist and his/her team will be present, and it will be possible to solicit the presence of other specialists, as well as observers from the manufacturer of the equipment and material used. My signature at the end of this consent form authorizes the participation of these professionals and grants them full access to records and medical information related to my case. I have also been informed that, should it be necessary, bed restraint may be performed, as prescribed by the physician, and that I will be under the surveillance of the nursing staff.

I further declare that I have had the opportunity to read this document carefully and also to listen attentively to the physician’s reading of all the information presented herein, and I was able to clarify all of my doubts regarding the risks involved and the possible consequences of not performing the suggested treatment, as well as the possible existence of alternative procedures, and my doubts have been satisfactorily addressed and answered. I am aware that there is a possibility of alteration of the original procedure over the course of the aforementioned procedure and/or new procedure or surgery for the sake of continuity of treatment in a different region from the one originally designated, which, if applicable, will be evaluated, decided upon, and performed, by the medical professional in charge, with which I now agree and hereby authorize.

All my questions having been answered and having understood the procedure in and of itself, as well as its real repercussions, I sign this CONSENT FORM, in the presence of Dr. ................................................................................................................, who, in this act, represents the Hemodynamic Medical Service, together with 2 (two) witnesses, expressly and voluntarily declaring my authorization for the procedure of angioplasty with carotid stent implantation, consenting, furthermore, to the performance of the proposed procedure as well as its preparatory acts; the physicians are hereby authorized to performed additional procedures and therapeutic interventions that may become necessary due to unforeseen conditions which may occur during the course of the procedure, remaining always at the discretion and judgment of the attending physician. Finally, I consent to the eventual possibility of a blood transfusion, should this procedure be directly linked to the treatment mentioned in this consent and authorization form or resulting from it.

If, during or immediately after the performance of the procedure which I am to undergo, I am not in full possession of my physical and/or mental abilities, I authorize my legal representative ................................................................................................................................, nationality ...................................................., marital status ......................................................., profession ....................................................., identity document .................................................., issued by ................................., CPF ..............................................................., resident of ......................................................................................................................................, city ................................................................, state ................., date of birth ........../........../.........., parents’ names........................................................................................................................................., relationship to patient ............................................................, to make decisions in my name.

In this manner, I offer my full and free consent, and I authorize the performance of the procedure of angioplasty with carotid stent implantation, explained herein, to be administered by the signing physician and his/her related medical team.

In conclusion, I certify, that I have signed two copies of the present Form, one of which has been given to me.

**Table:**

Center Location: ..........................................	
Date: ........../........../..........	
Patient's Name: ......................................................	Signature: ..................................................
	
Representative: ............................................	Signature: ...................................................
	
Physician's Name: ...................................................	CRM nº.: ..........................
Signature: ..................................................	
	
Witnesses:	
Name: ........................................................ Name: ........................................................	

## Annex 14

**FREE AND INFORMED CONSENT AND AUTHORIZATION FORM FOR PERFORMANCE OF THE PROCEDURE OF ANGIOPLASTY WITH RENAL ARTERY STENT IMPLANTATION**

By the present Consent and Authorization Form, I, ........................................................................................................, nationality ................................................, a legal adult and able, marital status .........................................................., profession ....................................................., identity document ......................................................., issued by ......................................., CPF ...................................................., resident of ..........................................................................................................................................., city .............................................................., state ....................., date of birth ........../........../.........., parents’ names ........................................................................................................................................., hereby declare that I have received from the Hemodynamics Service, here represented by the physician fully identified below, explanations and warnings concerning the procedure solicited by my clinical physician and that the discussion regarding the nature and extent of the actions necessary for its execution is registered below.

I further declare that it has been explained to me that the procedure angioplasty with renal artery stent implantation will be performed in the hemodynamics laboratory of this hospital institution (corporate name .............................................................................................., CNPJ/MF no. ...................................headquarters....................................................................) and that it consists of a puncture in the skin of the patient in order to introduce special catheters, using iodized contrast, with the administration of local anesthesia, sedation, or general anesthesia, at the attending physician’s discretion.

The following text has been read and explained to me in more accessible language by the signing physician: the aim of this procedure is to treat obstructions or blocks in the renal artery, clearing the obstruction by inflating a small balloon catheter in the site of the block, followed by implantation of a metallic mesh called a stent, via percutaneous access. The procedure is performed by means of a puncture in the femoral artery in the inguinal region, into which a catheter is introduced close to the diseased renal artery. X-ray emitting equipment and iodized contrast are used to identify the block in the artery. A very small guidewire will cross the obstruction, allowing for positioning of the balloon catheter in the block. Subsequently, a stent will be positioned and released in the site. Finally, renal arteriography will be performed as final angiographic control.

I declare that, in the manner explained to me by the physician, I am perfectly able to understand what the procedure which I am to undergo will comprise.

I declare that I am aware that the intended purpose of performing the forenamed procedure may not be achieved, even though the physician and his/her team adopt the best techniques and employ all of the scientific means and resources available.

I am also aware that the procedure involves risks, and I have received all the pertinent information regarding possible complications due to known and unknown causes, including death, stroke, myocardial infarction, cardiac arrhythmia, acute pulmonary edema, anaphylactic shock, varying types and degrees of infection, allergies and/or reactions to contrast, bleedings, hematomas, renal insufficiency, vascular and hemodynamic complications, perforation of cardiac chambers or vessels, and loss of limbs and/or their function, in addition to the risks inherent in anesthesia and the use of diverse instruments and equipment itself. It has also been explained to me that these adverse reactions and infrequent, occurring in less than 2% of cases, but they may be aggravated when associated with other patient personal factors, which include underlying diseases, previous heart surgery, prior history of allergies, uncontrolled arterial hypertension, tobacco use, alcoholism, diabetes, obesity, renal insufficiency, cerebrovascular disease, prolonged hospitalization, liver failure, heart disease, atherosclerotic disease, cancer, severe malnutrition, and advanced age, which are the most common.

It has also been explained to me that the catheters and prostheses used are subjected to previous tests, but that they may present defects or even fracture, causing adverse reactions and varying types and degrees of injury, including the possibility of requiring surgery to remove them.

It has been reiterated to me that no guarantees or assurances are given with respect to the results expected from the proposed procedure. I am aware that, in executing the proposed procedure, the hemodynamicist and his/her team will be present, and it will be possible to solicit the presence of other specialists, as well as observers from the manufacturer of the equipment and material used. My signature at the end of this consent form authorizes the participation of these professionals and grants them full access to records and medical information related to my case. I have also been informed that, should it be necessary, bed restraint may be performed, as prescribed by the physician, and that I will be under the surveillance of the nursing staff.

I further declare that I have had the opportunity to read this document carefully and also to listen attentively to the physician’s reading of all the information presented herein, and I was able to clarify all of my doubts regarding the risks involved and the possible consequences of not performing the suggested treatment, as well as the possible existence of alternative procedures, and my doubts have been satisfactorily addressed and answered. I am aware that there is a possibility of alteration of the original procedure over the course of the aforementioned procedure and/or new procedure or surgery for the sake of continuity of treatment in a different region from the one originally designated, which, if applicable, will be evaluated, decided upon, and performed, by the medical professional in charge, with which I now agree and hereby authorize.

All my questions having been answered and having understood the procedure in and of itself, as well as its real repercussions, I sign this CONSENT FORM, in the presence of Dr. ................................................................................................................, who, in this act, represents the Hemodynamic Medical Service, together with 2 (two) witnesses, expressly and voluntarily declaring my authorization for the procedure of angioplasty with renal artery stent implantation, consenting, furthermore, to the performance of the proposed procedure as well as its preparatory acts; the physicians are hereby authorized to performed additional procedures and therapeutic interventions that may become necessary due to unforeseen conditions which may occur during the course of the procedure, remaining always at the discretion and judgment of the attending physician. Finally, I consent to the eventual possibility of a blood transfusion, should this procedure be directly linked to the treatment mentioned in this consent and authorization form or resulting from it.

If, during or immediately after the performance of the procedure which I am to undergo, I am not in full possession of my physical and/or mental abilities, I authorize my legal representative ................................................................................................................................, nationality ...................................................., marital status ......................................................., profession ....................................................., identity document .................................................., issued by ................................., CPF ..............................................................., resident of ......................................................................................................................................, city ................................................................, state ................., date of birth ........../........../.........., parents’ names........................................................................................................................................., relationship to patient ............................................................, to make decisions in my name.

In this manner, I offer my full and free consent, and I authorize the performance of the procedure of angioplasty with renal artery stent implantation, explained herein, to be administered by the signing physician and his/her related medical team.

In conclusion, I certify, that I have signed two copies of the present Form, one of which has been given to me.

**Table:**

Center Location: ..........................................	
Date: ........../........../..........	
Patient's Name: ......................................................	Signature: ..................................................
	
Representative: ............................................	Signature: ...................................................
	
Physician's Name: ...........................	CRM nº.: ..........................
Signature: .........................................	
	
Witnesses:	
Name: ........................................................ Name: ........................................................	

## Annex 15

**FREE AND INFORMED CONSENT AND AUTHORIZATION FORM FOR PERFORMANCE OF THE PROCEDURE OF ANGIOPLASTY WITH LOWER LIMB ARTERY STENT IMPLANTATION**

By the present Consent and Authorization Form, I, ........................................................................................................, nationality ................................................, a legal adult and able, marital status .........................................................., profession ....................................................., identity document ......................................................., issued by ......................................., CPF ...................................................., resident of ..........................................................................................................................................., city .............................................................., state ....................., date of birth ........../........../.........., parents’ names ........................................................................................................................................., hereby declare that I have received from the Hemodynamics Service, here represented by the physician fully identified below, explanations and warnings concerning the procedure solicited by my clinical physician and that the discussion regarding the nature and extent of the actions necessary for its execution is registered below.

I further declare that it has been explained to me that the procedure angioplasty with lower limb artery stent implantation will be performed in the hemodynamics laboratory of this hospital institution (corporate name .............................................................................................., CNPJ/MF no. ...................................headquarters....................................................................) and that it consists of a puncture in the skin of the patient in order to introduce special catheters, using iodized contrast, with the administration of local anesthesia, sedation, or general anesthesia, at the attending physician’s discretion.

The following text has been read and explained to me in more accessible language by the signing physician: the aim of this procedure is to treat obstructions or blocks in the arteries of lower limbs, via percutaneous access, clearing the obstruction by inflating a small balloon catheter in the site of the block, followed by implantation of a metallic mesh called a stent, via percutaneous access. The procedure is performed by means of a puncture in the femoral artery in the inguinal region (groin), into which a catheter is introduced. X-ray emitting equipment and iodized contrast are used to identify the block in the artery. A very small guidewire, specific to this procedure, will cross the obstruction and allow for positioning and inflation of the balloon catheter in the block. Subsequently, a stent will be positioned and released in the same site. Once the device has been successfully released, final angiographic control is performed; the catheter is removed, and occlusive dressing is applied.

I declare that, in the manner explained to me by the physician, I am perfectly able to understand what the procedure which I am to undergo will comprise.

I declare that I am aware that the intended purpose of performing the forenamed procedure may not be achieved, even though the physician and his/her team adopt the best techniques and employ all of the scientific means and resources available.

I am also aware that the procedure involves risks, and I have received all the pertinent information regarding possible complications due to known and unknown causes, including death, stroke, myocardial infarction, cardiac arrhythmia, acute pulmonary edema, anaphylactic shock, varying types and degrees of infection, allergies and/or reactions to contrast, bleedings, hematomas, renal insufficiency, vascular and hemodynamic complications, perforation of cardiac chambers or vessels, and loss of limbs and/or their function, in addition to the risks inherent in anesthesia and the use of diverse instruments and equipment itself. It has also been explained to me that these adverse reactions and infrequent, occurring in less than 2% of cases, but they may be aggravated when associated with other patient personal factors, which include underlying diseases, previous heart surgery, prior history of allergies, uncontrolled arterial hypertension, tobacco use, alcoholism, diabetes, obesity, renal insufficiency, cerebrovascular disease, prolonged hospitalization, liver failure, heart disease, atherosclerotic disease, cancer, severe malnutrition, and advanced age, which are the most common.

It has also been explained to me that the catheters and prostheses used are subjected to previous tests, but that they may present defects or even fracture, causing adverse reactions and varying types and degrees of injury, including the possibility of requiring surgery to remove them.

It has been reiterated to me that no guarantees or assurances are given with respect to the results expected from the proposed procedure. I am aware that, in executing the proposed procedure, the hemodynamicist and his/her team will be present, and it will be possible to solicit the presence of other specialists, as well as observers from the manufacturer of the equipment and material used. My signature at the end of this consent form authorizes the participation of these professionals and grants them full access to records and medical information related to my case. I have also been informed that, should it be necessary, bed restraint may be performed, as prescribed by the physician, and that I will be under the surveillance of the nursing staff.

I further declare that I have had the opportunity to read this document carefully and also to listen attentively to the physician’s reading of all the information presented herein, and I was able to clarify all of my doubts regarding the risks involved and the possible consequences of not performing the suggested treatment, as well as the possible existence of alternative procedures, and my doubts have been satisfactorily addressed and answered. I am aware that there is a possibility of alteration of the original procedure over the course of the aforementioned procedure and/or new procedure or surgery for the sake of continuity of treatment in a different region from the one originally designated, which, if applicable, will be evaluated, decided upon, and performed, by the medical professional in charge, with which I now agree and hereby authorize.

All my questions having been answered and having understood the procedure in and of itself, as well as its real repercussions, I sign this CONSENT FORM, in the presence of Dr. ................................................................................................................, who, in this act, represents the Hemodynamic Medical Service, together with 2 (two) witnesses, expressly and voluntarily declaring my authorization for the procedure of angioplasty with lower limb artery stent implantation, consenting, furthermore, to the performance of the proposed procedure as well as its preparatory acts; the physicians are hereby authorized to performed additional procedures and therapeutic interventions that may become necessary due to unforeseen conditions which may occur during the course of the procedure, remaining always at the discretion and judgment of the attending physician. Finally, I consent to the eventual possibility of a blood transfusion, should this procedure be directly linked to the treatment mentioned in this consent and authorization form or resulting from it.

If, during or immediately after the performance of the procedure which I am to undergo, I am not in full possession of my physical and/or mental abilities, I authorize my legal representative ................................................................................................................................, nationality ...................................................., marital status ......................................................., profession ....................................................., identity document .................................................., issued by ................................., CPF ..............................................................., resident of ......................................................................................................................................, city ................................................................, state ................., date of birth ........../........../.........., parents’ names........................................................................................................................................., relationship to patient ............................................................, to make decisions in my name.

In this manner, I offer my full and free consent, and I authorize the performance of the procedure of angioplasty with lower limb artery stent implantation, explained herein, to be administered by the signing physician and his/her related medical team.

In conclusion, I certify, that I have signed two copies of the present Form, one of which has been given to me.

**Table:**

Center Location: ..........................................	
Date: ........../........../..........	
Patient's Name: ....................................................	Signature: ..................................................
	
Representative: .........................................	Signature: ...................................................
	
Physician's Name: ...................................................	CRM nº.: ..........................
Signature: ..................................................	
	
Witnesses:	
Name: ........................................................ Name: ........................................................	

## Annex 16

**RECOMMENDATIONS FOR WRITING REPORTS IN HEMODYNAMICS AND INTERVENTIONAL CARDIOLOGY (MINIMUM REQUIREMENTS)^70,71^**

Reports in hemodynamics and interventional cardiology are individualized for each physician, hemodynamics service, and procedure performed. The continuous methodological and technological evolution in interventional cardiology leads to minimal uniformity in order to meet current expectations to facilitate comprehension of each procedure by the patient, the hospital, and the source of payment.

The composition of a report in hemodynamics and interventional cardiology includes the following minimal elements:

1. Patient’s general information: name, sex, date of birth, day, time, hospital registration, procedure number, source of payment, referring physician.
2. Clinical indication for the procedure.
3. Procedure performed (e.g., left catheterization, coronary cineangiography and left ventriculography; coronary angioplasty with stent implantation; alcohol septal ablation; temporary pacemaker implantation, etc.).
4. Participating physicians: auxiliary physicians and anesthetist.
5. Technique utilized:
  - Access route.
  - Material utilized.
  - Catheter size (French scale).
  - Number of projections (usual or otherwise).
  - Technique for vascular hemostasis.
  - Use of vasoactive drugs or IIb/IIIa platelet antagonists.
6. Procedure findings:
  - Manometry (invasive pressure of chambers studied).
  - Oximetry, calculation of cardiac output, flows, and resistances, in indicated cases.
  - Coronary cineangiography.
  - Left and right ventriculography and ascending aortography.
  - Coronary, structural, extracardiac, congenital intervention.
  - Other results of complementary imaging methods and vascular/coronary physiology performed.
7. Procedure time, radiation dose, type and volume of contrast use in the patient.
8. Complications.
9. Final conclusion on the findings.
10. Comments: note difficulties with projections, different catheter exchanges, angiography of the access site, change of initial access for another.

## Annex 17

STRUCTURED FORM | TRAINEE

**Table:**

1. Training Center: ....................................................................................
2. Person Responsible for the Training Center: .....................................................
3. Date: ......../......../........

• Does your Training Center possess the infrastructure recommended in the latest Guidelines on Professional Quality of the Brazilian Society of Hemodynamics and Interventional Cardiology (SBHCI)?
_____ Yes _____ No

• During your first year, did you receive training in radiation protection, vascular accesses, complications and management thereof, diagnostic percutaneous procedures, and low complexity coronary interventions?
_____ Yes _____ No

• During your second year, did you receive training in high complexity coronary interventions, adjuvant methods to coronary cineangiography/intervention (FFR, iFR, ICUS, or OCT), and management of structural cardiopathies?
_____ Yes _____ No

• During your entire training period, did you perform, as a first operator under supervision, at least 400 diagnostic cardiac catheterizations and 200 coronary angioplasties?
_____ Yes _____ No

• Did you regularly (at least twice per month) participate in reunions in conjunction with the clinical team and the heart surgery team (heart team)?
_____ Yes _____ No

• Did you have regular courses that covered at least 70% of the Theoretical Program suggesting in the latest Guidelines on Professional Quality of the SBHCI?
_____ Yes _____ No

• Assign a score between 0 and 10 to your Training Center and make suggestions for improvement for the SBHCI to forward to your Training Center coordinator:
Note:..............
Suggestions:................................................................................................................................................................. .............................................................................................................................................................................................. .............................................................................................................................................................................................. .............................................................................................................................................................................................. .............................................................................................................................................................................................
